# COVID‐19 field instruction: Bringing the forests of British Columbia to students 8,000 km away

**DOI:** 10.1002/nse2.20040

**Published:** 2021-03-10

**Authors:** Patrick D. Culbert

**Affiliations:** ^1^ Dep. of Forest and Conservation Sciences Forest Sciences Centre 3613, Univ. of British Columbia 2424 Main Hall Vancouver BC V6T 1Z4 Canada

## Abstract

Field instruction is a crucial component of natural sciences education. The COVID‐19 pandemic has shifted many university courses to an online format, significantly impacting field instruction. FRST 350, *Foundational Field School*, is an 8‐day University of British Columbia Forestry field course taught to incoming transfer students from partner universities in China. In August 2020, I taught this course online to students studying remotely. In re‐developing the course, I spent 9 days in the field filming high definition (HD) video, 360° video, and 360° photography to best recreate the course in a short time‐frame. A 360° video records omnidirectionally, allowing viewers to “look around” in all directions, resulting in a highly immersive experience. Students expressed favorable opinions of the course, especially traditional HD and 360° video. Students generally preferred HD videos over 360°, though this was due mostly to the high bandwidth needed for 360° video and the fact that core course content was primarily conveyed as HD videos (in recognition of bandwidth challenges), with supplementary 360° videos. Students favorably noted the interactivity and immersive feel of 360° videos and photographs. This technology is financially and logistically feasible for use in a natural sciences course. Instructors engaged in online field instruction should weigh the strengths and weaknesses of various technologies, including 360° video, when determining how to best meet their learning objectives.

**Abbreviations**
BC: British Columbia
BEC: biogeoclimatic ecosystem classification
FRST 350: Foundational Field School 8‐day course
HD: high definition
LMS: learning management system
MKRF: Malcolm Knapp Research Forest
UBC: University of British Columbia
VR: virtual reality

## 1 INTRODUCTION

In March 2020, the emerging COVID‐19 pandemic (Fauci, Lane, & Redfield, 2020) forced universities to quickly pivot from face‐to‐face to online instruction. With negligible preparation time, faculty were typically unable to deliberately transform their courses using sound online pedagogy (Carey, 2020; Gardner, 2020). As the pandemic continues and many universities remain at least partially online, faculty are seeking improved ways to employ online teaching for courses traditionally held face‐to‐face. Field instruction is a critical component of university education in the natural sciences (Fleischner et al., 2017). Abruptly converting face‐to‐face field instruction to an online format in response to COVID‐19 has been a significant challenge (Barton, 2020). Instructors perceive negative effects related to learning outcomes, with a shift toward more instructor‐centered, rather than student‐centered approaches, and instructors have concerns about equity, especially regarding student access to technology (Barton, 2020).

The University of British Columbia's (UBC) Faculty of Forestry offers a number of field courses among several degree programs. FRST 350, *Foundational Field School*, is an 8‐day field course designed specifically for participants in the Faculty of Forestry's 3+2 program. In this program, Chinese students take 3 years of courses at a partner university in China, including some short, intensive courses taught by visiting UBC faculty. Students then transfer to UBC and complete two additional years of coursework to receive their UBC degree. For many 3+2 students, FRST 350 is their first course at UBC. The course runs in late August, immediately preceding the fall term. FRST 350 is a bridge course, combining concepts and skills from second‐year courses and introducing topics from upcoming third‐year courses. The objectives of FRST 350 are:

- Objective 1: To integrate and apply topics covered in second‐year courses regarding plants, soils, ecology, and basic management of BC forests
- Objective 2: To give hands‐on experience in field skills such as navigation and mensuration
- Objective 3: To introduce upcoming third‐year topics such as silviculture, insects and disease, and forest operations
- Objective 4: To assist students in their transition and acculturation to UBC

Given the COVID‐19 pandemic (Fauci et al., 2020) in late‐spring 2020, the decision was made to cancel all UBC undergraduate forestry summer field courses. Because FRST 350 is a key transition course for newly arrived 3+2 students, I felt complete cancellation would be quite detrimental. Because 3+2 students receive transfer credit for core second‐year courses rather than taking them at UBC, this field course helps them catch up to their new non‐transfer peers. In particular, 3+2 students are at a disadvantage in third‐year courses because their non‐transfer peers have already spent extensive time learning in local BC forests. Additionally, given travel restrictions and online‐only instruction, all 3+2 students would be remotely taking their fall UBC courses while still in China. Therefore, knowledge and context of local ecosystems would be especially crucial to students’ success in their other courses. With limited time, it was not feasible to fully re‐create FRST 350 in an online format. I sought to create a pared‐down online course for August 2020, followed by an abbreviated field course that focused on field skills in August 2021 (if possible). Of the course objectives listed above, the online version prioritized Objective 1 (applying topics from second‐year courses) and Objective 3 (introducing concepts to be covered in third‐year courses) and I focused my efforts on preparing materials that, based on my previous experience teaching the course, would yield the highest (learning) return on (material development time) investment. Course Objective 2 (hands‐on experience with field skills) was dropped as infeasible and Objective 4 (transition and acculturation) was not emphasized, although teams did get practice communicating in English about forestry through daily team reports and field notebooks, and they were encouraged to speak English while working within their teams. Conditions permitting, an abbreviated field excursion is planned for summer 2021 to complete the remaining course objectives.

**Core Ideas**

- The COVID‐19 pandemic has forced natural sciences field instruction to be taught online.
- Although online field instruction has shortcomings, many learning objectives may be achieved.
- 360° video and photography are feasible approaches for more realistic remote field instruction.

## 2 ONLINE FIELD INSTRUCTION

Field instruction is critical to education in the natural sciences (Fleischner et al., 2017). It is one of many forms of experiential education, where students learn by doing and then reflecting on their experience (Kolb, 2014). Though field instruction is usually taught face‐to‐face, virtual field experiences have been in use prior to the COVID‐19 pandemic. Virtual field trips, often delivered online, may include text, photographs, video, and other electronic resources (Woerner, 1999). Advantages over actual field excursions include low cost (Jacobson, Militello, & Baveye, 2009; Litherland & Stott, 2012), accessibility for students with disabilities (Elleven, Wircenski, Wircenski, & Nimon, 2006; Krzic, Wilson, & Hoffman, 2018), the ability to engage with locations where travel is not feasible (Jacobson et al., 2009), and high reusability (Tuthill & Klemm, 2002). A significant downside is limited interaction by the learner (Tuthill & Klemm, 2002), as interactive elements in the field are potentially infinite, whereas interactive elements in a virtual experience are limited to those developed by the content creator.

Video is an obvious medium for online delivery of field instruction, as even most mobile phones are now capable of recording reasonable‐quality video and audio, and a number of platforms simplify online hosting of video. In a survey of ecology and evolution instructors teaching during the spring 2020 pivot, many respondents noted an increased use of video, especially to replace field lectures and instructor demonstrations (Barton, 2020). One challenge with replacing field excursions with video is that it cannot fully replicate the experience of actually being in a field location. This is due in part due to the limited field of view provided to the user and the limited interactivity. Virtual reality (VR) technologies hold promise, although VR has not been widely adopted in teaching (Radianti, Majchrzak, Fromm, & Wohlgenannt, 2020). This is partly because of the high cost of developing VR resources and the costs of head‐mounted displays for viewing VR content, although accessibility is increasing with low‐budget adapters for mobile devices such as Google Cardboard (Radianti et al., 2020). There are, however, quite recent examples of VR use in undergraduate courses, such as enabling students to interact with a VR model of a cell to gain insight into structures and organelles that are otherwise difficult to visualize (Bennett & Saunders, 2019) or creating a VR version of a traditional in‐person organic chemistry laboratory with interactive 360° video (Dunnagan et al., 2020). This organic chemistry VR activity was also subsequently used in distance‐learning during the COVID‐19 transition to online courses, but limited student access to VR viewers or reliable, high‐speed internet access presented challenges for some students (Dunnagan & Gallardo‐Williams, 2020).

In particular, 360° video is a promising approach to developing VR resources for use in teaching. In 360° video, a camera with two or more lenses records in all directions simultaneously. Rather than a rectangular video, this in essence produces a sphere of video, allowing the viewer to change their view left, right, up, or down while the video plays (Snelson & Hsu, 2020). In contrast to other VR approaches, 360° video relies primarily on real‐world footage, not computer‐generated images (Snelson & Hsu, 2020). This is well‐suited to a field course that aims to represent actual environments rather than computer‐generated ones. Consumer‐grade 360° video cameras are available from manufacturers including GoPro, Insta360, and Ricoh starting at US$500. Video hosting platforms such as YouTube, Kaltura, and Vimeo support 360° videos. When viewed on a computer, users drag their mouse to change their orientation. On devices such as tablets or phones, views can be changed by tilting or moving the device. Head‐mounted devices may also be used for a more immersive experience.

FRST 350 is primarily a survey field course, introducing a range of topics, typically with short (10‐to‐15‐minute) field lectures followed by hands‐on activities. The course culminates with student teams spending a full day conducting mapping, measurements, and ecological assessment of a forest stand. One less tangible aim for the course is helping students develop an “eye” for the forest, becoming good observers and drawing inference about past forest stand history and future trajectories. To best accomplish this aim virtually, I chose 360° video as a medium of course delivery. However, these video files are quite large and require high bandwidth and computing power to view at high resolution. I therefore also produced traditional high‐definition (HD) video and 360° still photographs, a low‐bandwidth alternative that allows the user to “look around” a still photograph. Although video resources have been previously used in UBC forestry courses (e.g., Culbert, 2020), no course has used 360° video. I was therefore interested in students’ perceptions of the comparative value of traditional HD video, 360° video, and 360° photographs and their impressions of an online field course more generally. Therefore, upon the course conclusion, I administered an anonymous, voluntary student survey.

## 3 METHODS

### 3.1 Seeking and developing educational resources

With limited time, I first sought existing materials for use in the course. I used a website and video series (Hoffman, Krzic, Nashon, & Schmidt, 2017; Krzic et al., 2018) to give students the appropriate background in forest soils. I also re‐used a subset of videos on plant identification and ecological characteristics that I had produced previously (Culbert, 2020). In the face‐to‐face course, many activities were composed of a 10‐to‐20‐minute field lecture followed by an activity where students would further explore a concept or practice a skill. To recreate these approaches in video form, I recorded field lectures almost exclusively as traditional HD video, as these required less bandwidth and I could focus the students’ attention on a specific view. These videos tended to be longer and served as the primary course content. To recreate students’ ability to explore a site, most field lecture videos were accompanied by a shorter 360° video where I would elaborate a bit while moving around the site. As an alternative resource (primarily as a low‐bandwidth alternative to 360° video), I also typically took 360° photographs from several positions at a site. Because these were supplemental, these photographs were presented without narration or additional explanation.

Time limitations unfortunately precluded the development of extensive interactive activities, although I created a few. In these cases, I played the role of the teammate in the field, with students recording and analyzing data as they observed me gathering it. For example, to teach fixed‐area plot measurement, I filmed myself measuring the trees and calling the measurements back to the camera for students to record and perform the necessary calculations, which they reported in their online field notebook (video available at https://youtu.be/wqegaASU5EU).

### 3.2 Video production

**FIGURE 1:**
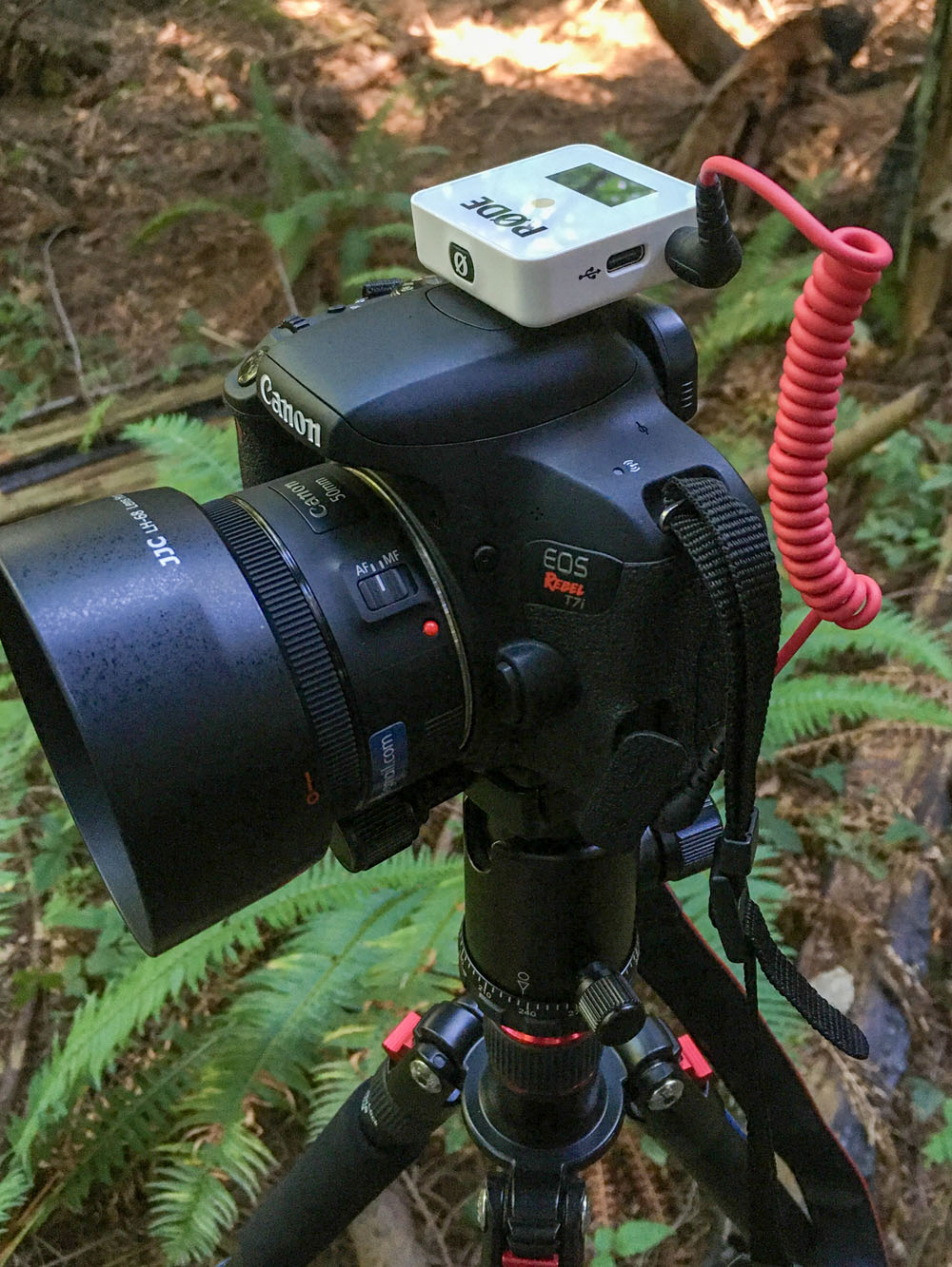
Tripod‐mounted Canon DSLR camera used for filming traditional high definition (HD) videos, with RØDE wireless microphone receiver attached

**FIGURE 2:**
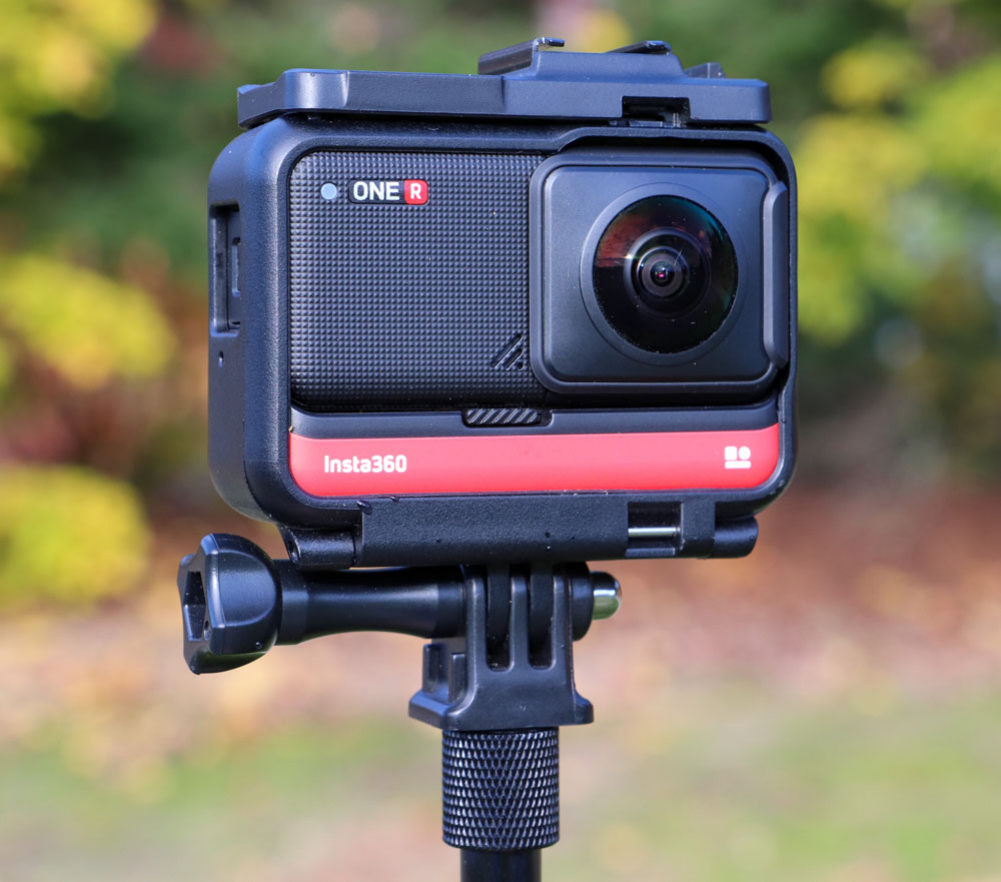
Insta360 ONE R 360° camera used for recording 360° videos and photographs. In addition to the fisheye lens pictured, the camera has an identical lens on the opposite side, to simultaneously record in all directions

I recorded traditional HD videos with a tripod‐mounted Canon T7i DSLR camera (Figure 1), capturing audio with a RØDE VideoMicro microphone or a RØDE Wireless Go transmitter/receiver with a RØDE Lavalier Go lapel microphone. Due to COVID restrictions, I filmed most videos solo, remotely operating the camera with an Apple iPhone or iPad. I recorded 360° photos and videos with an Insta360 ONE R (Figure 2). The 360° photos and videos are recorded at the same resolution, although the photos are much smaller files as they consist of a single image. I mounted this palm‐sized camera on either a handheld selfie‐stick or a tripod. I used either the camera's built‐in microphone or the RØDE Wireless Go and RØDE Lavalier Go lapel mic. The 360° video files are quite large, and the ability of a student to look around the scene was not always necessary to achieve a video's aims. In most filming locations, I therefore used multiple media. For a typical topic, the main “field lecture” video was recorded as a traditional HD video. A shorter, supplementary video was recorded in 360° to give students a more immersive experience and give better spatial context. I also took several 360° photographs at each site. These were supplementary and also served as an alternative 360° view of a location for students whose low internet bandwidth prevented them from viewing 360° videos.

I filmed on nine different days in July and August 2020 at multiple locations in BC's lower mainland: UBC's Malcolm Knapp Research Forest (MKRF), Pacific Spirit Regional Park, UBC Farm, and Skagit Valley Provincial Park. At MKRF I was joined for 2 days by Cheryl Power, assistant manager of the research forest, who appeared on‐camera to discuss ecology and forest management. Jeremy Watkins, assistant operations manager of the research forest, joined me on camera for 1 day to speak about forest operations and road development. I primarily edited traditional HD videos using Camtasia (Camtasia, 2020) and 360° videos using Insta360 Studio (Insta360 Studio, 2020). I also used Adobe Premiere Pro (Adobe Premiere Pro, 2020) when more advanced editing (especially of 360° video) was needed.

**FIGURE 3:**
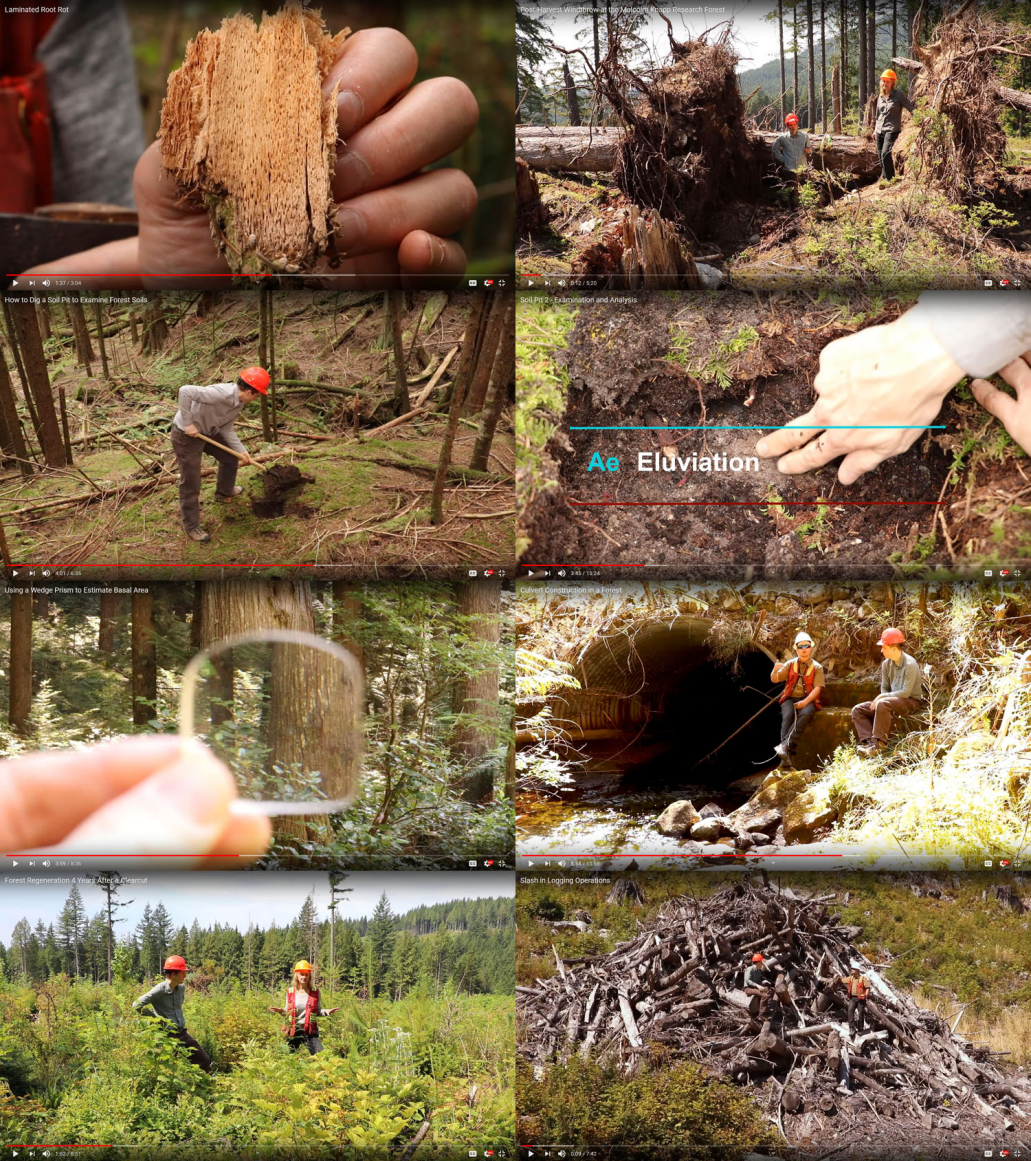
Screenshots from traditional high definition (HD) videos produced for FRST 350, Foundational Field School. Video topics represented are (clockwise from upper left) laminated root rot, windthrow, examining forest soil horizons, culvert construction, slash management, forest regeneration, forest measurements with a wedge prism, and digging a soil pit to examine forest soils

**FIGURE 4:**
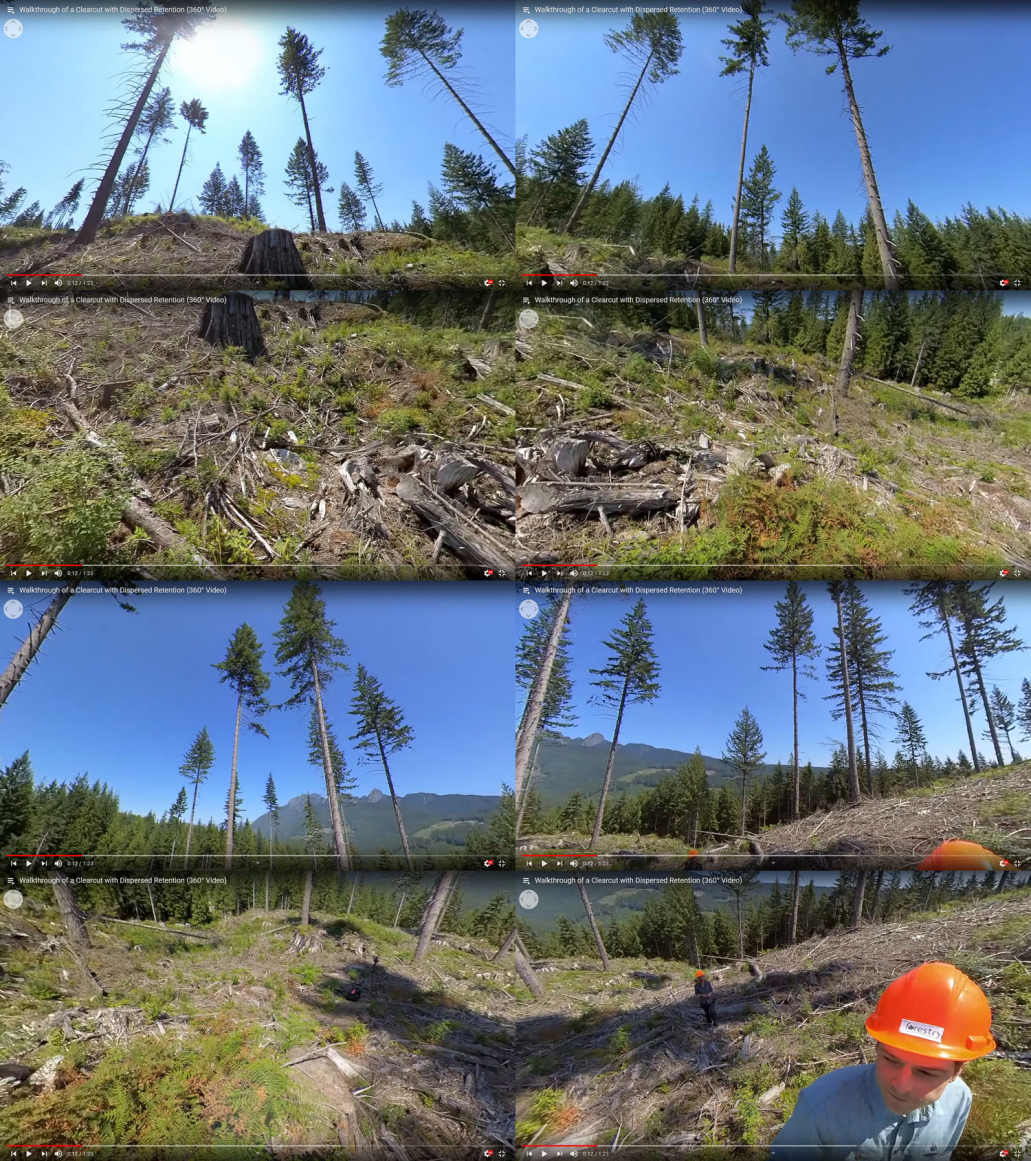
Multiple views of a single frame in a 360° video showing the results of a clearcut harvest with dispersed retention at Malcolm Knapp Research Forest. Each image shows a different possible view depending on the user's viewing orientation at that moment

**FIGURE 5:**
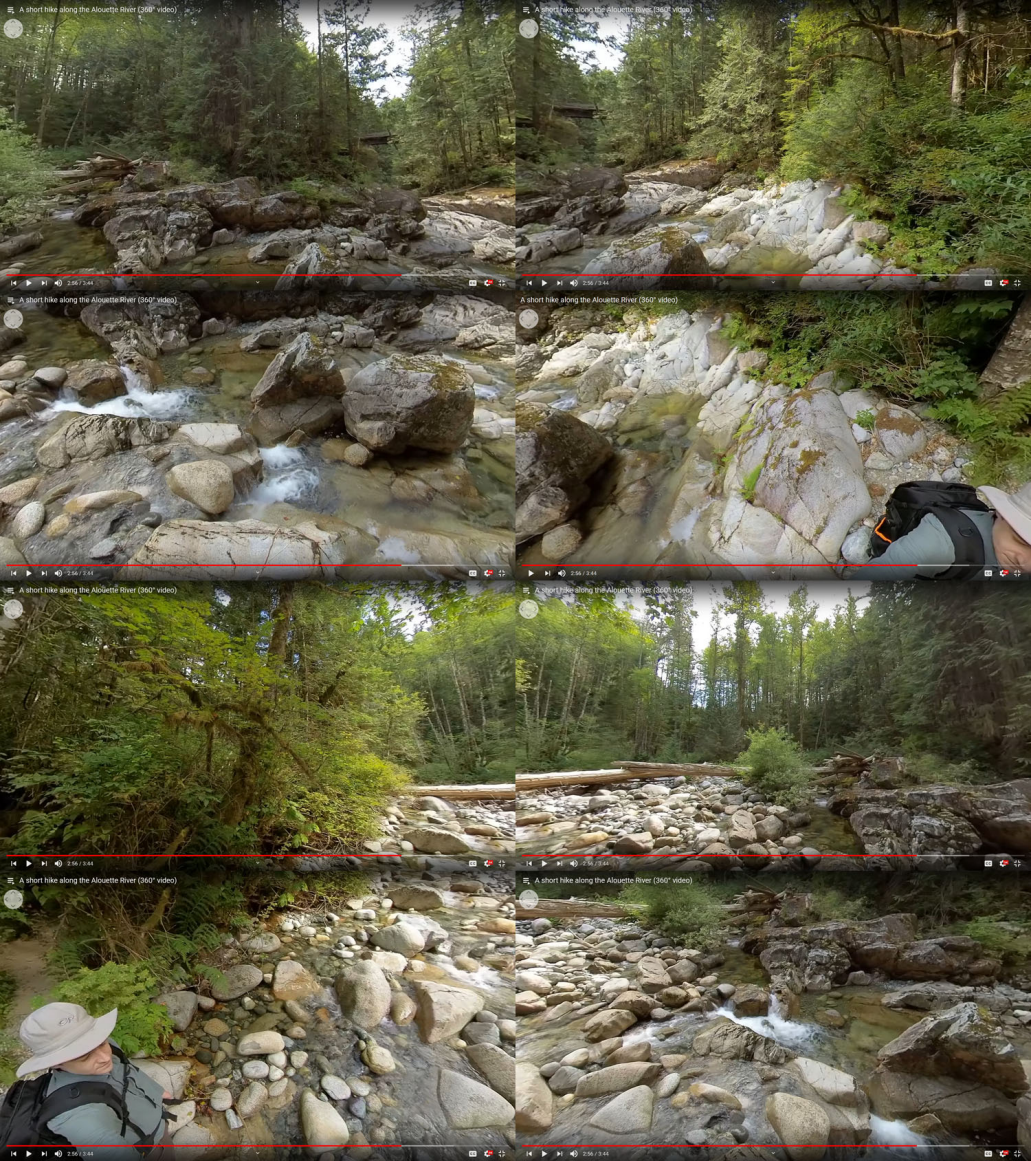
Multiple views of a single frame from a 360° video of the Alouette River in Malcolm Knapp Research Forest. Each image shows a different possible view depending on the user's viewing orientation at that moment

I produced a total of 113 new videos for this course, totaling just under 10 hours with an average video length of 5.25 minutes (Figures 3, 4, 5). This total excludes the existing plant identification and soil videos that were originally developed for other courses. Of the 113 videos, 45 were 360° videos and 68 were traditional, HD. I produced small sets of 360° photographs for 15 of the recording sites. I posted videos on YouTube for test viewing and the generation and editing of captions. Videos were also posted via Kaltura because YouTube is blocked in China, preventing student access without a virtual private network (VPN) connection. Unfortunately, UBC's implementation of Kaltura limited the resolution of the streaming 360° videos to 1,920 × 1,080 with a maximum bitrate of 5 Mbps. This is sufficient for traditional HD video but results in a significant loss of quality for 360° videos filmed at 5,760 × 2,880 resolution. Through the Kaltura platform, I did enable student download of the higher‐resolution videos for offline viewing. However, the files were quite large and lacked the closed captions that were viewable on the streaming videos.

### 3.3 Course structure

FRST 350 is normally taught exclusively in the field. The course is 8 full days with 2 days in on‐campus forests at UBC, a 2‐day field trip to dry forests in the BC Interior, and 4 days at Malcolm Knapp Research Forest. When taught face‐to‐face, students are assigned to teams of four, and they work almost exclusively in these teams, with rotating daily roles (team leader, data recorder, spokesperson, and devil's advocate). A large portion of students’ individual grades are determined by the quality of their field notebooks, where they write notes, summaries, and reflections on each activity. Grades also include a plant identification exam, individual oral exam, and oral and written presentations of each team's stand assessment.

Because the online format afforded greater flexibility, I extended the course to 2 weeks (without adding additional material) to reduce the daily pace. Student individual grades were based primarily on their digital field notebooks, maintained in Canvas, the learning management system (LMS) used by UBC. In addition to including notes, summaries, and reflections, the daily class instructions also included some specific questions related to the day's activities, all to be included in the field notebooks. Students were also graded on an online plant identification exam. The oral exam and team field assessment were not retained in this online version of the course (with the hope that these will be carried out at a later date when conditions allow an abbreviated in‐person field excursion). In Week 1 of the course, students individually worked through four self‐paced background modules: coastal trees and understory plants; soils; biogeoclimatic ecosystem classification (BEC) system; and tree measurements and forest inventory.

Week 2 began with a synchronous kick‐off meeting via videoconference. Like in the face‐to‐face course, instructor‐determined teams were formed, intentionally mixing students from different Chinese universities into gender‐balanced or female majority teams (as recommended by Dasgupta, Scircle, & Hunsinger, 2015). Within teams, students also rotated through the daily roles. In this week, students worked through a new module each day (see the Supplemental material for a sample module). Modules consisted almost exclusively of short videos. In their digital field notebook, students would write notes, reflections, and answers to specific questions posed within the module. Students were encouraged to work collaboratively with their teammates, although each student was responsible for their own field notebook. At day's end, the team spokesperson posted a brief daily report answering: (a) “What went well today?” (b) “What didn't?” (c) “What was the most interesting, surprising, or enjoyable thing you learned or did today?” and (d) “What do you still have questions about?” Outside of the team report, students were also encouraged to post their questions on the course discussion board.

The Week 2 daily modules were:

- Day 1: Forest Ecology in the Coastal Western Hemlock Zone
- Day 2: Forest Ecology, Forest Health, and Field Skills at Malcolm Knapp Research Forest
- Day 3: Silviculture and Forest Measurements at Malcolm Knapp Research Forest
- Day 4: Forest Operations at Malcolm Knapp Research Forest
- Day 5: A Visit to the Interior Douglas‐Fir BEC Zone and Site Classification at the UBC Farm Forest

Although students worked synchronously with their teams, they were asynchronous from me, as they were 15 time zones ahead. With this time difference, I began work each morning as the students ended their day. I would read and respond to the team reports and individual student questions and make final adjustments for the next day's material. In late afternoon, I would post the new material and a daily announcement so it would be ready as students began their day. I posted the announcement as text, but I also included short videos I had pre‐recorded in the field outlining that day's activities (view example at https://youtu.be/yHXKfMqubCo).

### 3.4 Survey

Upon completion of the course, I provided students with a voluntary, anonymous survey that asked for their views on the online field course, with a specific emphasis on the different technologies (traditional HD video, 360° video, and 360° photographs) used to present the course material (see the Supplementary material). Respondents were asked to rate their agreement or disagreement, on a 7‐point Likert scale, to the following statements:

- My internet connection speed was adequate for viewing the traditional (non‐360°) videos
- My internet connection speed was adequate for viewing the 360° videos
- The traditional (non‐360°) videos improved my understanding of the course material
- The 360° still photographs improved my understanding of the course material
- The 360° videos improved my understanding of the course material
- When viewing the 360° videos, I was able to easily follow along and change my view to the object that Dr. Culbert was explaining
- I frequently changed my view to look around while viewing 360° videos

The survey also asked students: (a) if they made use of captions, and if so, did they aid understanding of the course material; (b) to rank the three technologies used with regard to their effectiveness in improving understanding of course material and to explain those rankings; (c) whether videos were typically downloaded or streamed, and on which type of device they were viewed; (d) what were the advantages and disadvantages of each of the three technologies used; (e) what aspect(s) of the course they liked most and least and how the course could be improved; and (f) what advice they would give to instructors teaching an online field course. The survey was administered with Qualtrics (Qualtrics, 2019) and was approved by the UBC Behavioral Research Ethics Board (H20‐02629).

## 4 RESULTS

### 4.1 Survey results

Of the 31 students in the course, 17 completed the survey, yielding a response rate of 55%.

### 4.2 Comparison of traditional HD video, 360° video, and 360° photographs

Students agreed that each of the three technological approaches improved their understanding of the course material but more strongly favored traditional HD videos (Likert‐scale interpolated median = 1.85) than 360° photographs (2.56) or 360° video (2.57) (Table 1). Students indicated they were able to follow along in the 360° videos, and nearly all students made use of their ability to “look around” in those videos (Table 1). With regard to effectiveness in improving understanding of the course material, students decisively ranked the traditional HD videos most effective, followed by 360° videos, then 360° photographs (Table 2). The justification of the rankings, however, indicates that the student preferences were influenced more by how the technologies were employed in the course, and not specifically on the merits of the technology. Students noted that the traditional HD videos were more explanatory in nature and conveyed most of the important concepts, whereas the 360° videos were much larger files and thus streamed with lower quality. However, students did report that the 360° videos gave a better impression of being in the forest, or that with a good internet connection, they were superior. One student explained, “Traditional videos explain concepts in more detail, which is the basis for my understanding of knowledge. On this basis, 360° videos or pictures are used to help me deepen my understanding. However, my 360° video has a lag, so the viewing effect is not very good.” Another student stated, “The traditional video is the fundamental knowledge of the course, while I can use 360° photo to digest the knowledge and relate to reality. I think 360° video can assist me to relate my knowledge to reality but it is optional.”

**TABLE 1:** Student agreement with statements regarding the use of video in the online version of the field course. Likert scale level of agreement: 1 = strongly agree, 2 = agree, 3 = somewhat agree, 4 = neither agree nor disagree, 5 = somewhat disagree, 6 = disagree, 7 = strongly disagree

Survey statement	Level of agreement							Interpolated median
	Strongly agree		↔			Strongly disagree		
	1	2	3	4	5	6	7	
The traditional (non‐360°) videos improved my understanding of the course material.	5	10	2	–	–	–	–	1.85
The 360° still photographs improved my understanding of the course material.	4	4	8	1	–	–	–	2.56
The 360° videos improved my understanding of the course material.	3	5	8	1	–	–	–	2.57
When viewing the 360° videos, I was able to easily follow along and change my view to the object that Dr. Culbert was explaining.	4	7	6	–	–	–	–	2.14
I frequently changed my view to look around while viewing 360° videos.	5	5	4	2	1	–	–	2.2
My internet connection speed was adequate for viewing the traditional (non‐360°) videos.	1	1	9	2	4	–	–	3.22
My internet connection speed was adequate for viewing the 360° videos.	–	2	9	2	2	2	–	3.22
The video subtitles (closed captions) aided my understanding of the course material.	11	1	–	–	–	–	–	1.05

*Note*. For each question, the median response is underlined.

**TABLE 2:** Student ranking of the usefulness of each of the technological approaches in improving their understanding of the course material

Medium	Most effective (1)	Neutral (2)	Least effective (3)	Mean rank
Traditional videos	11	2	–	1.15
360° Photographs	–	3	10	2.77
360° Videos	2	8	3	2.08

When asked for advantages of traditional HD video, students nearly universally noted it was easier to follow the instructor, and these videos focused more closely on the main course content (e.g., “By explaining it in detail, it makes people understand it faster,” “It may contain more traditional knowledge which is useful for exam…”). In addition, several noted the file sizes were smaller so they were easier to download, and the image quality is better than 360° videos when bandwidth is limited. For disadvantages, students observed that it was a less realistic view of the forest compared to 360° video, and their view was limited (e.g., “not that flexible,” “We cannot see the whole view of one location,” “If there is an image, I can only view it in 2D form. It is somehow limited to view all aspects.”)

This mirrored student‐perceived advantages of 360° video and 360° photographs. Students appreciated the more holistic view of their surroundings (e.g., “I can see the whole environment,” “I feel like standing in the forest”) and the more interactive nature (e.g., “We can see where we want to see,” “I can explore the surroundings myself”) Disadvantages of 360° video were related to large file size and bandwidth issues (e.g., “too big and quality is low if you watch it online,” “connection issues when streaming”). These were not issues with the 360° photographs, as those files are quite small comparatively; however, students commented that they wanted more guidance in the viewing of these photographs (e.g., “they are silent, without instructors’ words and thoughts,” “I don't know what to do sometimes”).

Students mostly viewed streaming videos, although some downloaded the files and played them locally (Table 3). Students primarily viewed videos on laptop or desktop computers (13 used laptop computers, 2 used desktop computers, and 2 used tablets). Of 17 respondents, 12 reported making use of video captions. Of those, 11 strongly agreed and 1 agreed with the statement, “The video subtitles (closed captions) aided my understanding of the course material” (Table 1).

**TABLE 3:** Mode of video viewing (streaming vs. downloading and playing locally) primarily used by students

Medium	Download	Streaming
Viewing traditional videos	4	13
Viewing 360° Videos	6	11

### 4.3 General feedback on course

When asked which course aspect they liked the most, student responses were quite varied. A number noted the videos (e.g., “Even I can't go to the forest myself, I can view the forest more vividly! That is beyond my expectation!” “When instructor brought a camera and went into forests to show us how to work in it”). One student noted that as an English‐language learner, they appreciated the captions and their ability to watch the videos multiple times. Another liked that they did not actually need to go into the field. For aspects of the course they liked the least, most students cited bandwidth issues while a few noted the difficulties of online learning generally. Prompted for suggestions for this course specifically and online field courses generally, students suggested solving technical challenges, giving a lighter workload or reduced pace, shorter videos, and more live sessions.

## 5 DISCUSSION

Field instruction is a critical component of natural science education (Fleischner et al., 2017). The transition to online teaching due to the COVID‐19 pandemic has posed serious pedagogical challenges to teaching overall (Gardner, 2020) and to field instruction specifically (Barton, 2020). FRST 350, *Foundational Field School* is an 8‐day field course for incoming Chinese transfer students shortly after their arrival at UBC Forestry. In response to the pandemic, in August 2020, I taught the course in an online format. To recreate a field course remotely, I heavily relied on traditional HD video, 360° video, and 360° photography. Students expressed favorable opinions of these media but noted challenges, especially related to internet bandwidth. My experience and students’ feedback give insight for those remotely teaching field courses or field activities.

### 5.1 Student perceptions of an online field school

Students expressed favorable opinions of the course, while noting inherent challenges of an online field course. As one student said, “I still think the best way for a field trip is to go by ourselves. But this online course is great under such circumstance!” Students expressed support for the use of video, with 7 of 13 survey respondents mentioning video as an aspect of the course they liked the most. The most frequently cited challenge was poor internet connectivity. This especially impacted the 360° videos, as those files are quite large with image quality degrading substantially over slow connections. Students also suggested reducing the workload and giving more guidance to some activities. For example, a student suggested, “Can provide some learning target before watching the video.” This is similar to Hoffman et al. (2017) where a student noted it was sometimes difficult to identify important points from an explanatory video.

### 5.2 Online field school insights and recommendations

From the instructor perspective, it was challenging to estimate student workload, especially with a new mode of delivery, technical challenges, and varying levels of student English proficiency. Like in a face‐to‐face field course, flexibility is important, as is good communication between students and instructor. Through students’ daily team reports, it was clear they were struggling to keep pace. Midway through Week 2, I offered the option of a 1‐day “pause,” extending the overall course length by a day, allowing a day to catch up. Students were strongly in favor. As one later noted, “I understand that while giving online courses, there will me [sic] more materials for us to learn. But sometime the workload is overwhelming. So I think the mid‐break is necessary for us to keep up with the course.”

The digital field notebook fulfilled its purpose by incentivizing engagement without burdening students with quizzes and exams. Though summarizing and organizing ideas is an important role of the notebook, I especially value it for student reflection, as reflection as an integral part of the experiential learning process (Association for Experiential Education, 2020; Kolb, 2014; Moon, 2013). This course also represents a large transition for these students, with adjustment to new language, culture, and teaching approaches. An opportunity to reflect on this is important.

A favorite aspect of the face‐to‐face version of the course is the frequent formal and informal interaction with students. This was difficult in an asynchronous course. I worked hard to establish instructor presence though. I was personally on camera in almost all videos and spoke directly to students. I also posted a course introduction video prior to the start of the course (viewable at https://youtu.be/PNS3y1xwH1k) and included daily video announcements. Asynchronous video lessons and video communications can improve students’ feelings of connectedness to their instructor (Borup et al., 2012; Martin et al., 2018). The 15‐hour time zone difference also allowed me to respond to every team's daily report and individual student questions by the time they began class the next day.

My major regret was limited time to develop interactive activities. The time needed to design these activities well is much greater than the time to record a field lecture and pose a few questions to students; thus, I focused my energy on the latter approach. This led to a more passive experience than students would have had in the field. Given time, I would have developed activities where students “collected data” from photographs or video, or activities (like the fixed area plot exercise mentioned earlier) where students observe me collecting data and analyze those data in their teams.

### 5.3 Suggestions regarding video use

Students expressed an overall preference for the traditional HD video, but their comments indicate this is because I used that format to convey fundamental course material, whereas 360° video was used for shorter companion videos. Students did appreciate 360° videos for the ability to look around the forest and have an immersive experience. Because they were unable to visit the forest in person, this was especially important to me. In initial planning, I anticipated filming most video in 360°. However, during proof‐of‐concept tests it became apparent that the file sizes would be an obstacle. At the best resolution (5.7k), the insta360 ONE R captures video at 100 Mbps, or 750 MB per minute. This can be reduced through compression, but file size is still an issue, especially for students with low‐bandwidth connections (e.g., Dunnagan & Gallardo‐Williams, 2020). I therefore selected traditional HD video as my primary medium, reserving 360° video for when I wanted students to have an immersive view with the ability to look around. As a further complication, UBC's implementation of Kaltura limits video resolution to about 25% the native resolution of the 360° video. As a workaround, I made the higher‐resolution 360° videos available for direct download and encouraged students to do so. However, only 6 of 17 survey respondents reported downloading (rather than streaming) these videos, so most students were watching low‐resolution 360° videos. This was reflected in some comments about the lower visual quality. Many students expressed appreciation for the immersive and interactive character of the 360° videos, so this technology is still valuable given the drawbacks. Instructors must consider their learning objectives and potential technical limitations when choosing a medium. In my case, a mix of video types worked well.

**FIGURE 6:**
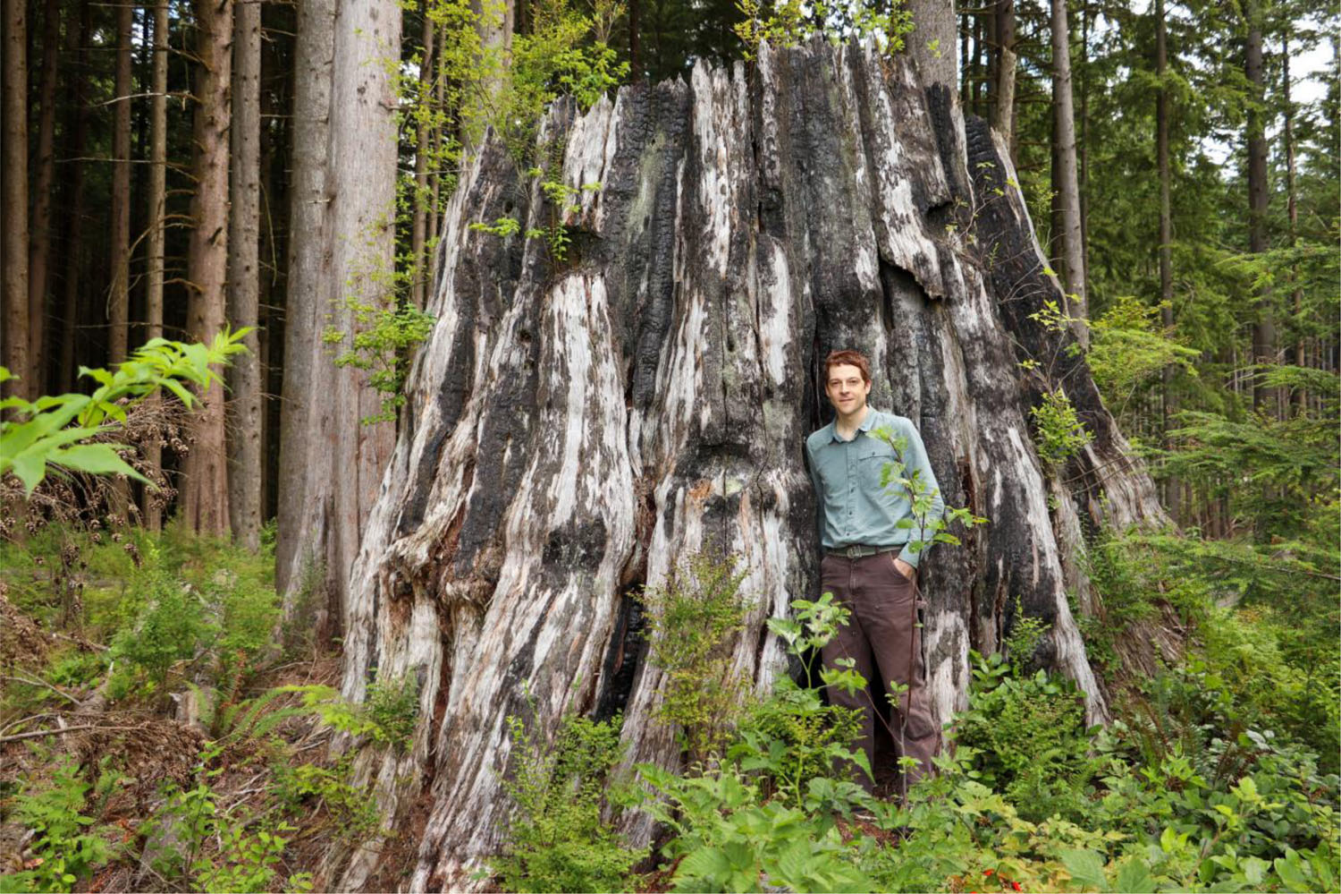
I recorded impromptu videos when encountering interesting examples in the field. Here I recorded a video after coming across a 3.5 meter‐diameter western redcedar (*Thuja plicata* Donn ex D. Don) stump harvested about 100 years earlier

I often teach opportunistically in the field (Ison & Bramwell‐Lalor, 2020; Steinert, Basi, & Nugus, 2017). A student or I will sight something interesting or unusual, and we will take time to discuss it. Fortunately, this also worked in a virtual context. During filming, I periodically encountered something unplanned, for example, a 3.5‐meter‐diameter western redcedar (*Thuja plicata* Donn ex D. Don) stump remaining from the original cutover 100 years ago, that warranted an explanation to students. In these cases, I simply set up my gear and filmed an impromptu video (Figure 6). I incorporated some of these videos as core content and shared others as “just for fun” viewing.

One aspect where I feel the videos and photos fell short was interactivity. It is technologically possible to add interactive elements to these media (e.g., Dunnagan et al., 2020). For example, I could have asked students to identify the plants in a 360° photograph, and when a given plant is clicked, the species name, or further information would be displayed. Similarly, directional buttons can be added to link videos or photos together, allowing students to virtually “tour” an area by choosing which direction to “walk.” Given the large amount of content required for this field course and the limited development time, it was only feasible to produce straightforward videos and photos with limited embellishment. With available time and resources, I recommend adding interactive elements to these media if they aid in holding the viewer's attention and advance the learning objectives.

Once familiar with the video equipment, I was able to film in a time‐efficient manner. Rather than full scripts, I used outlines of key points, and most videos covered topics I was comfortable speaking extemporaneously about. This unscripted approach supported time‐efficient production and short, conversational‐style videos, although scripted videos may have been more concise. The two‐person, interview‐style video format worked especially well. Prior to filming, I would discuss with the interviewee what I wanted to cover, and during the shoot I would prompt them with questions. Through questions, I could then guide the interviewee to key points or clarification of words or ideas in which the students were unlikely to be familiar.

Given that all students were English language learners, their preference for captions was expected. However, even when students are native English speakers and not hard‐of‐hearing, they may still value captions (Culbert, 2020; Hoffman et al., 2017). English language learner students also benefit from being able to pause a video to translate a term or to re‐watch portions of videos they did not understand, an option not available in live instruction. YouTube's auto‐captioning of videos had high accuracy, but all captions required manual editing, especially for jargon or scientific terms. Good sound quality, such as from a lapel microphone, improved auto‐captioning accuracy.

A significant advantage of virtual field trip content is its reusability (Tuthill & Klemm, 2002) and portability. As I produced these materials, I kept in mind other courses for which specific videos would be suited. As a survey field course, many of the FRST 350 videos are useful in other forestry courses, and some were used in at least three other UBC forestry courses in fall 2020. I made all videos freely available on YouTube (https://www.youtube.com/c/PatrickCulbertUBCForestry), and a number of instructors from other universities have included some of these videos in their courses.

## 6 CONCLUSION

I am hopeful that this pandemic will soon end, and we can return to field instruction as before. However, even after in‐person field instruction resumes, when it supports my learning objectives, I will incorporate virtual field experiences into my non‐field courses. This will give students a taste of the field even when a field trip is not feasible (Ramasundaram, Grunwald, Mangeot, Comerford, & Bliss, 2005; Stott, Nuttall, & McCloskey, 2009), and these virtual excursions are highly accessible to all students (Elleven et al., 2006). Even after this field course resumes in person, I will consider continuing to use some videos as supplementary or pre‐class material to give students relevant background knowledge while leaving more time in the field for hands‐on activities. I have found traditional HD video, 360° video, and 360° photography to all be useful approaches to virtually bring the forest to the students, especially for quickly recreating field demonstrations or brief field lectures. Although highly interactive activities (simulating hands‐on field activities) are feasible with these technologies, they require additional planning and development time. Of the technologies considered for this course, my opinion is that 360° video provides the most immersive and realistic viewer experience, even though large files and high bandwidth requirements are notable drawbacks. For instructors developing remote field instruction, I recommend piloting different approaches and technologies, weighing their strengths and shortcomings, and ultimately making decisions based on which approach is best suited to supporting students in achieving a course's learning objectives.

## CONFLICT OF INTEREST

The author declares no conflict of interest.

## Supporting information

Supplementary material

Supplementary material
