# Supplementary material for: COVID‐19 field instruction: Bringing the forests of British Columbia to students 8,000 km away
Source: Natural Sciences Education. 2021 Mar 10;50(1):e20040. doi: 10.1002/nse2.20040 (PMC7995167; doi:10.1002/nse2.20040)
Supplement: Supplementary file 1 — Supplementary material [file NSE2-50-e20040-s002.pdf]

Day 1: Forest Ecology in the Coastal Western Hemlock Zone

- Field Notebook Instructions. Day 1: Forest Ecology in the Coastal Western Hemlock Zone

Module Introduction

- Morning Welcome - Day 1
- Welcome to Pacific Spirit Park
- Coastal Western Hemlock BEC Zone
- CWH - Trees

An Ecological Tour of Pacific Spirit Park

- CWH in Pacific Spirit Park
- Becoming an Ecological Detective
- Pacific Spirit Park - Walk
- Plant ID Walk

Supplemental Information

- Coastal Western Hemlock Zone

Field Notebook Instructions. Day 1: Forest Ecology in the Coastal Western Hemlock Zone

For this module, I would like you to create 3 pages for your field notebook:

1. CWH - BEC Zone
2. Becoming an Ecological Detective
3. Pacific Spirit Park Walk

Note that for the **CWH - Trees (https://canvas.ubc.ca/courses/54569/pages/cwh-trees)** and **Plant ID Walk (https://canvas.ubc.ca/courses/54569/pages/plant-id-walk)** , you don't need to make a specific entry in your field notebook, but you should use what you learn here to add to or revise your previous field notebook entries on plants and trees.

Be sure to answer the relevant questions in your field notebook.

Morning Welcome - Day 1

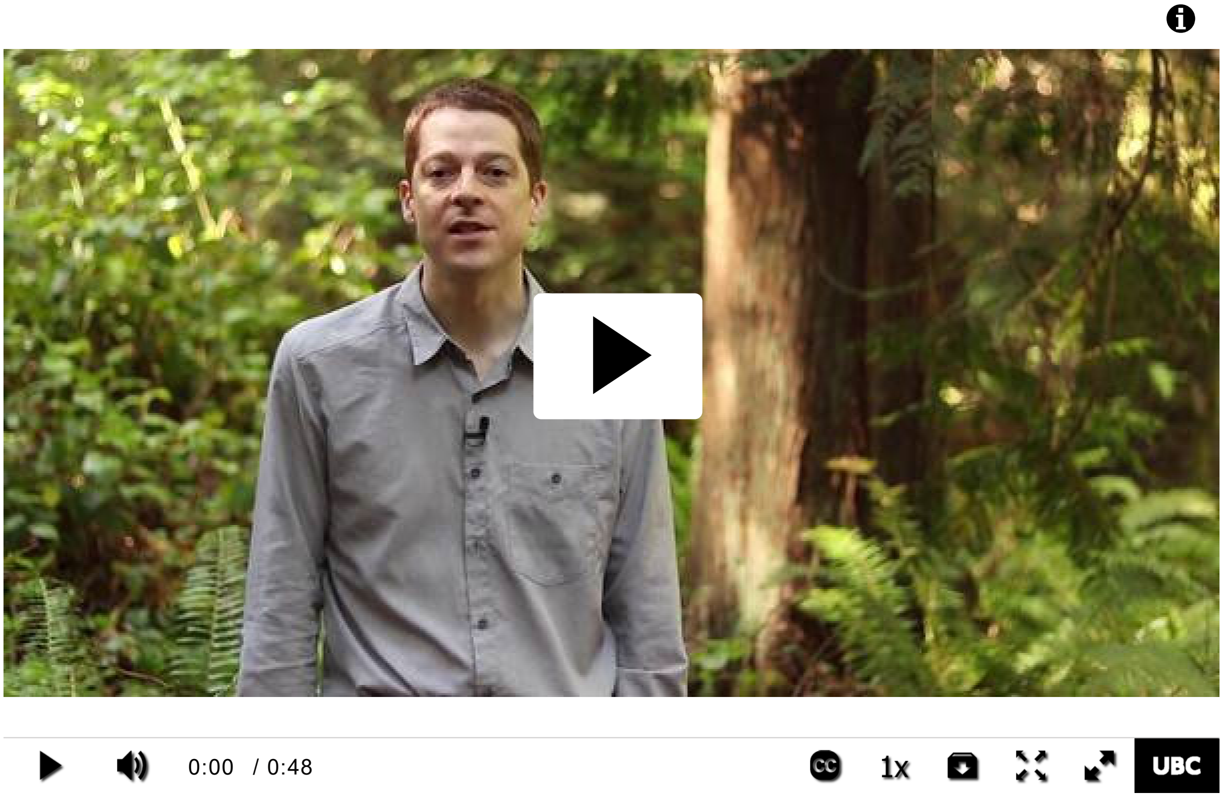

Welcome to Pacific Spirit Park

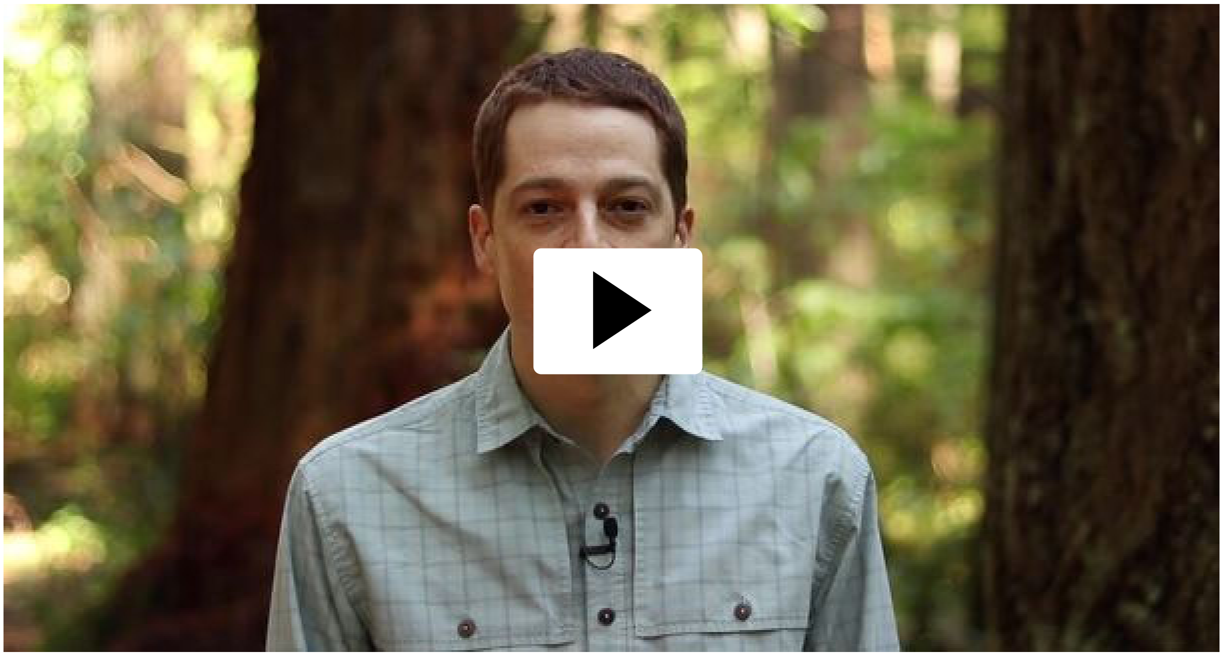

0:00 / 0:36 1x UBC

Coastal Western Hemlock BEC Zone

CWH - Overview

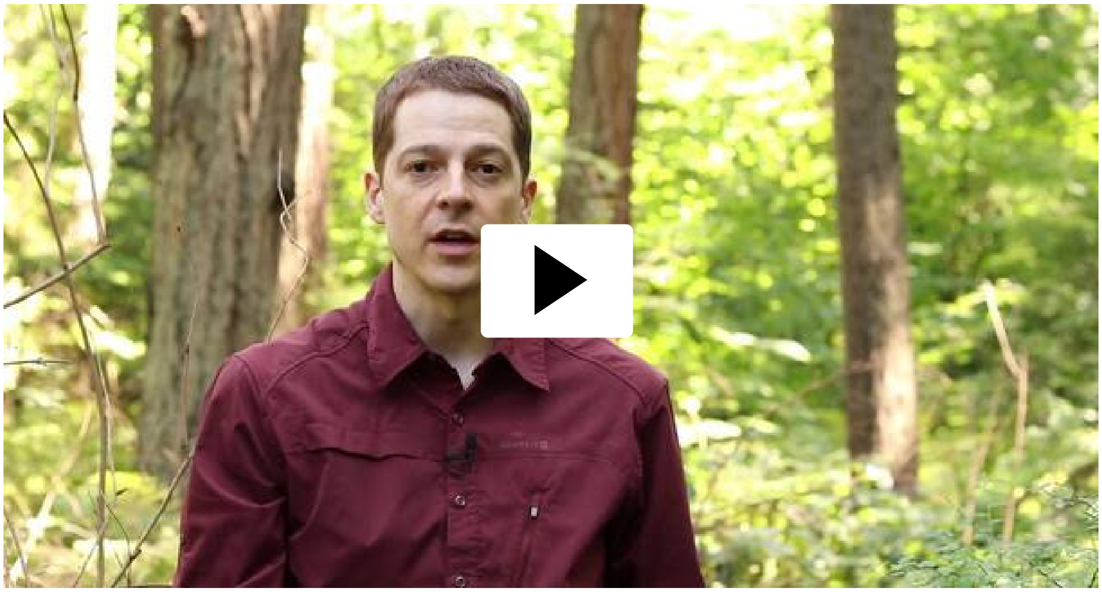

0:00 / 4:37 1x UBC

CWH - Disturbance

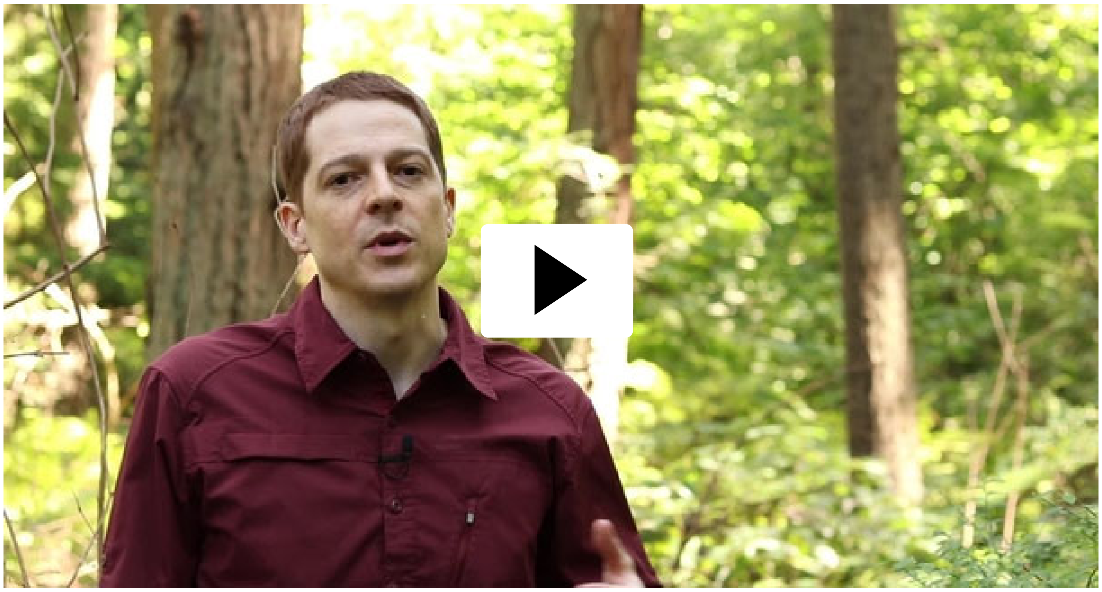

0:00 / 3:17 1x UBC

Field Notebook Questions:

1. Give a general description of the climate of the Coastal Western Hemlock Zone.
2. Name two important early-successional species in the CWH.
3. Name two important late-successional tree species in the CWH.
4. Describe the disturbance regime of the CWH Zone.

CWH - Trees

Key Tree Species in the CWH

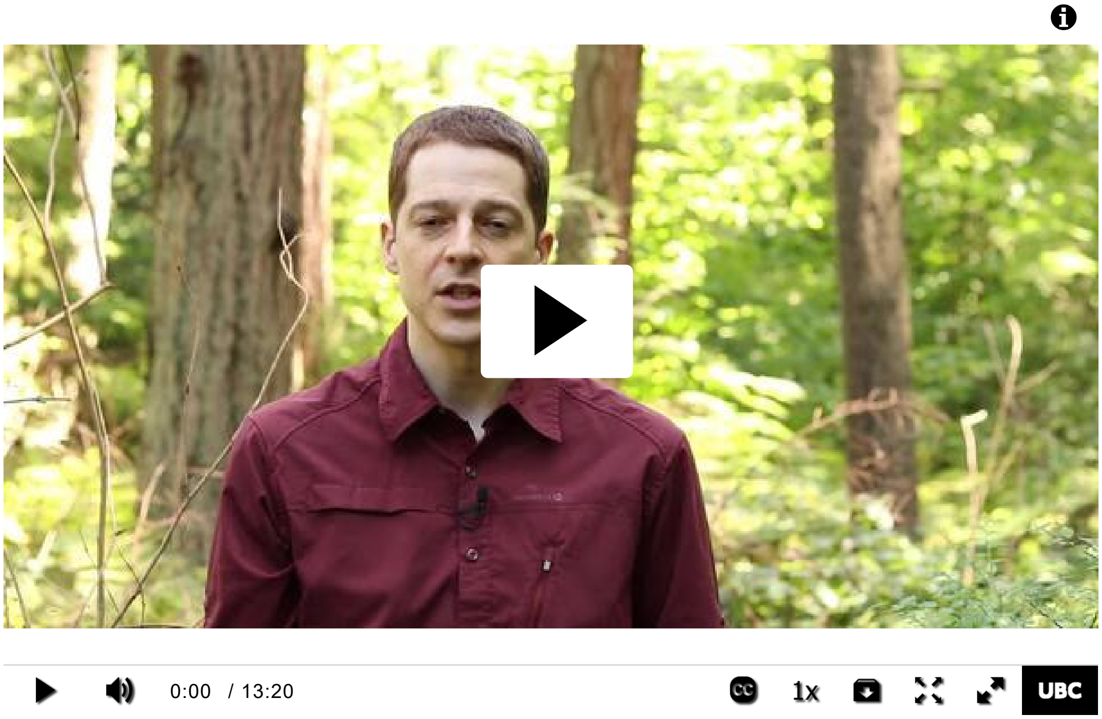

Identifying Trees by Bark

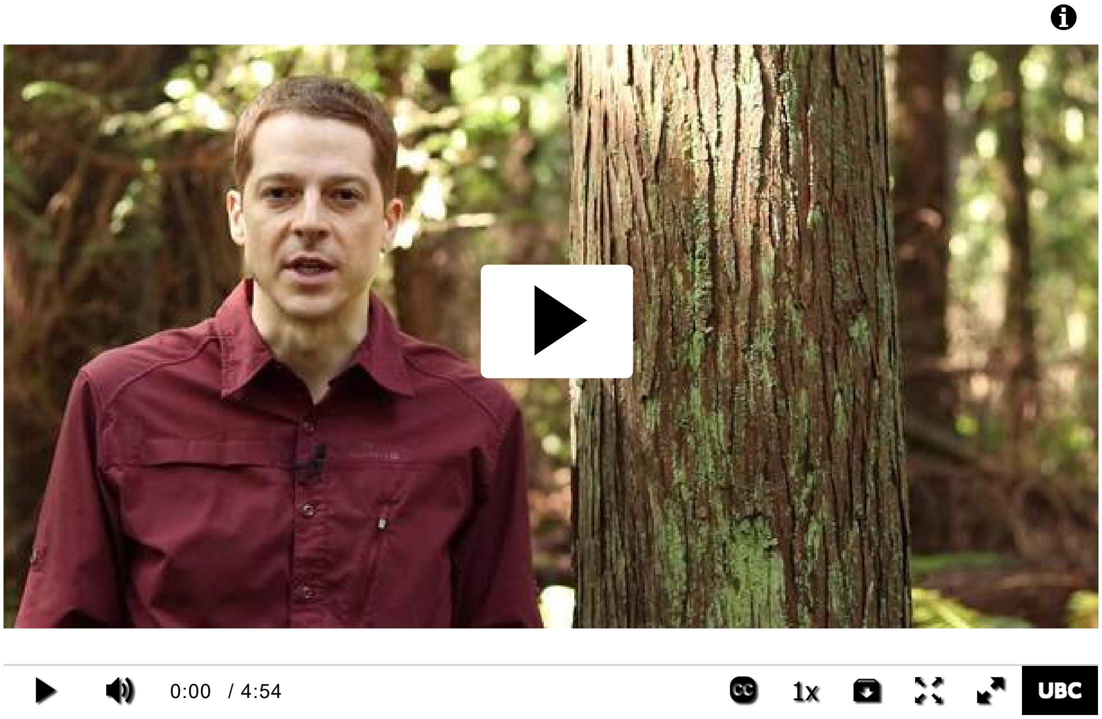

CWH in Pacific Spirit Park

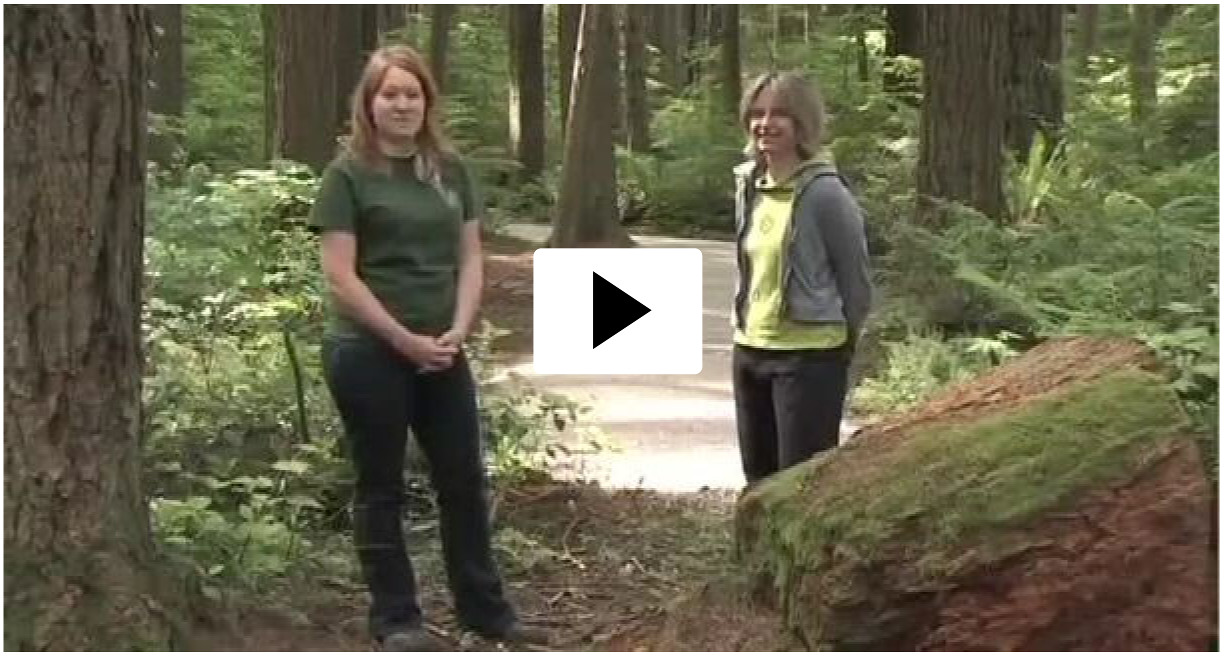

0:00 / 10:33

1x

UBC

Becoming an Ecological Detective

Ecological Detective - Part 1: Observation (360 Video)

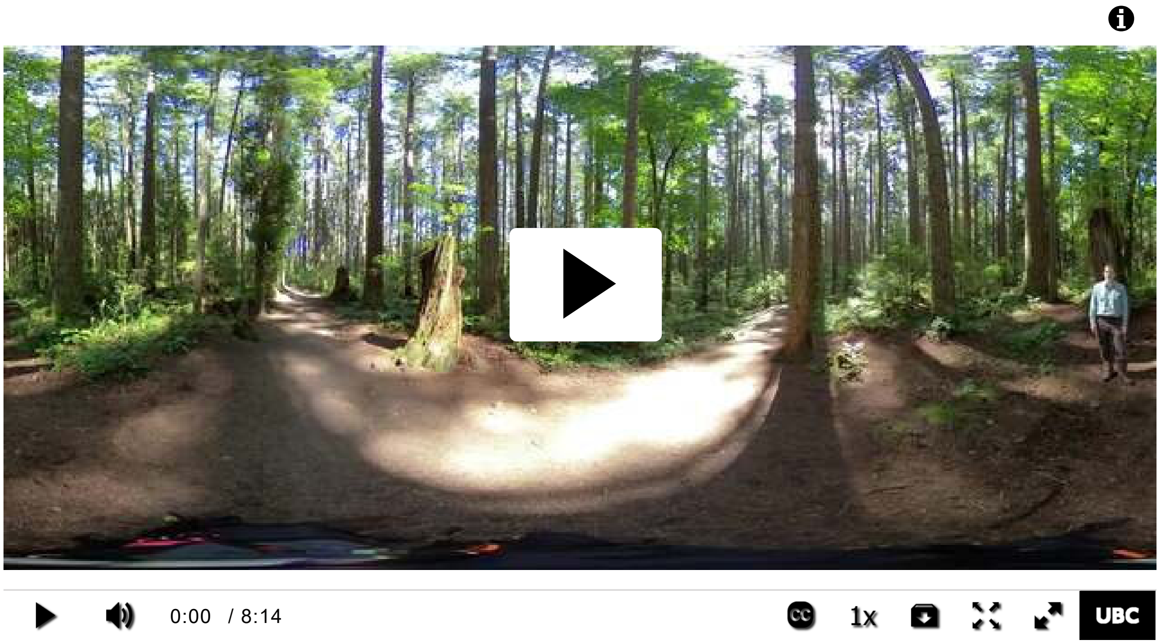

Ecological Detective - Part 2: Inference (360 Video)

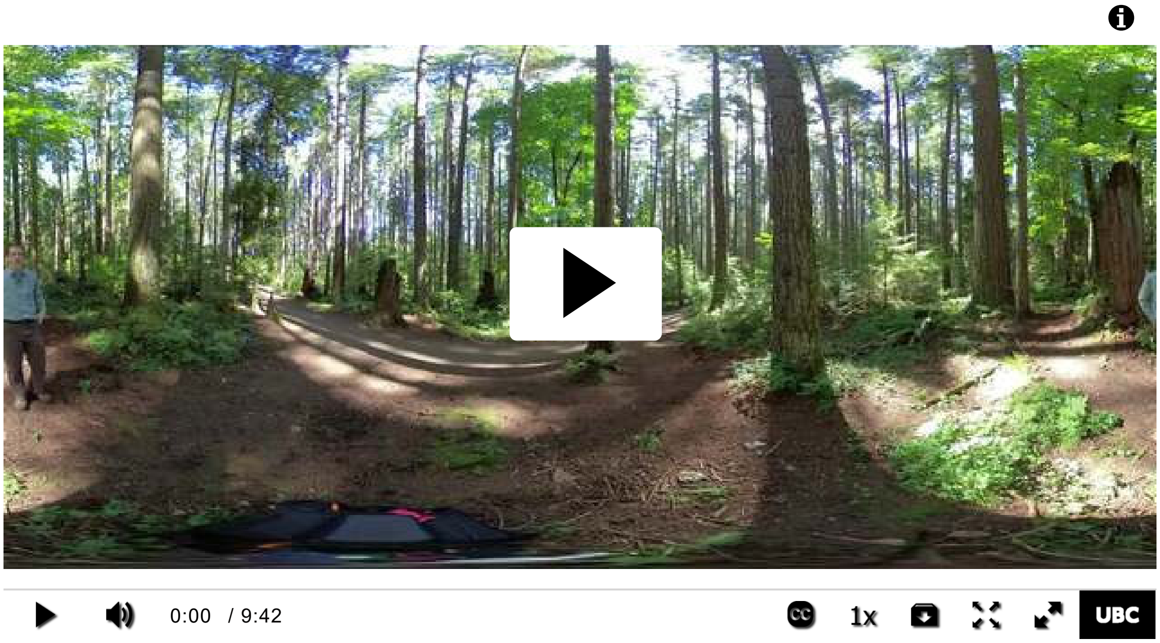

Ecological Detective - Forest Disturbance

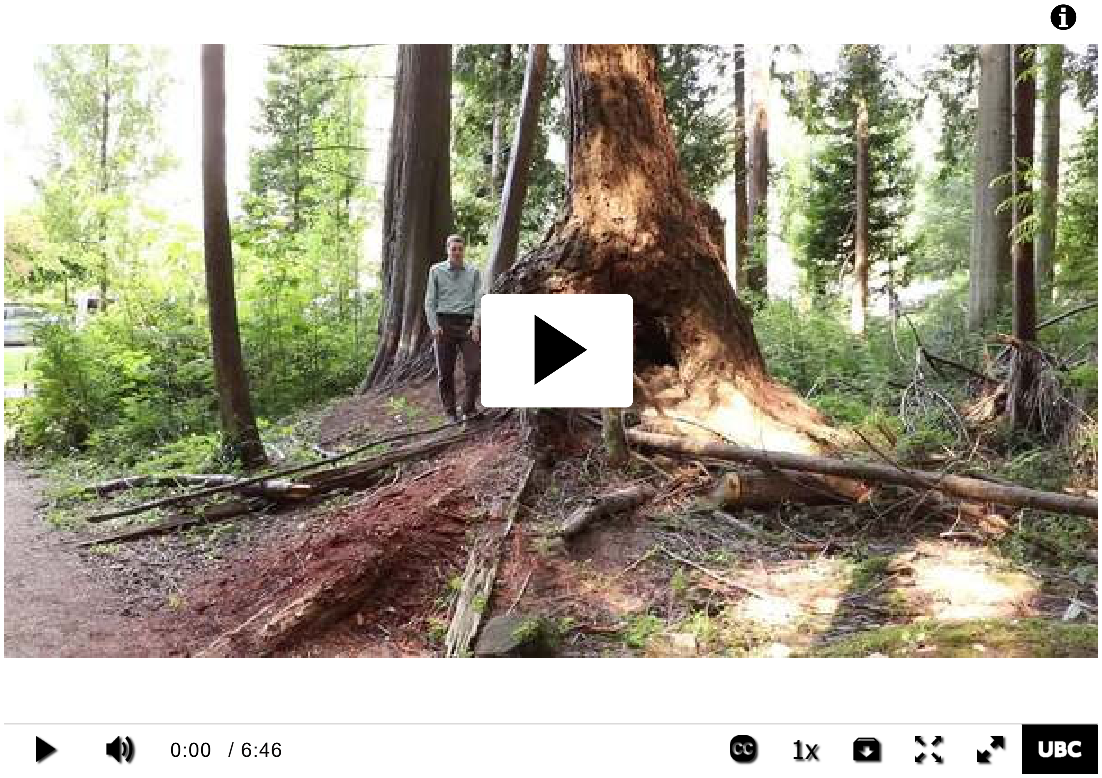

Questions for Field Notebook:

1. Describe the process of being an ecological detective.
2. What are some things you might observe in the forest that tells you about that stand's history?
3. What are some things you might observe in the forest that tells you about that stand's future?

Pacific Spirit Park - Walk

Bigleaf Maple Stump Sprout

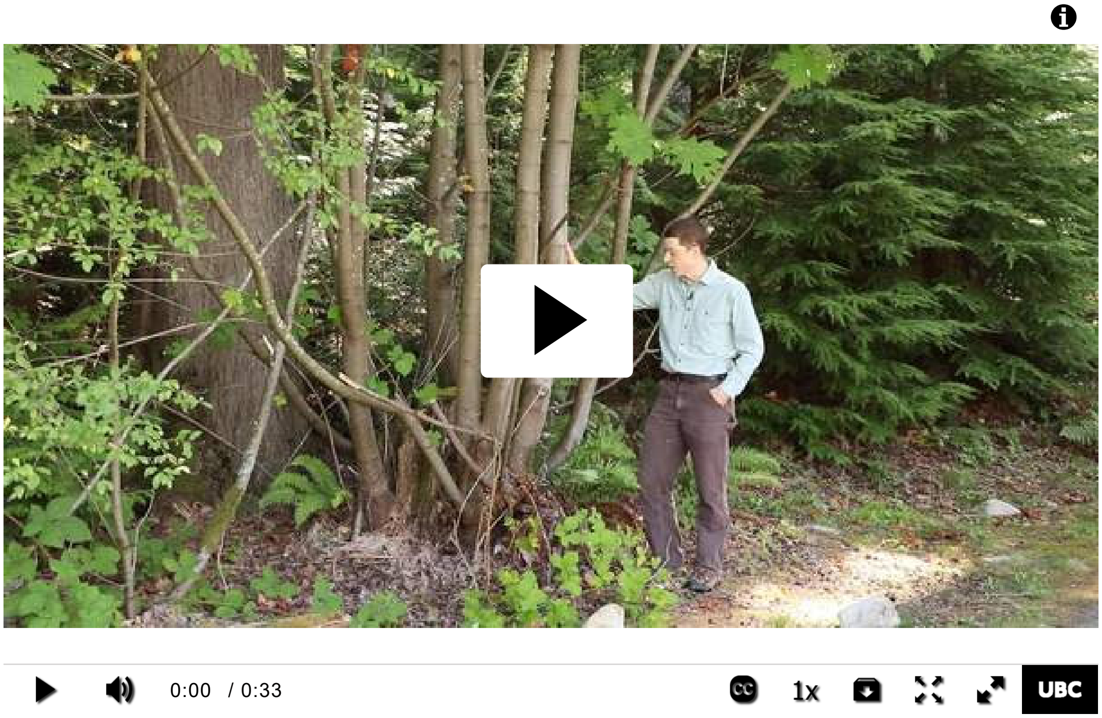

A Living Stump

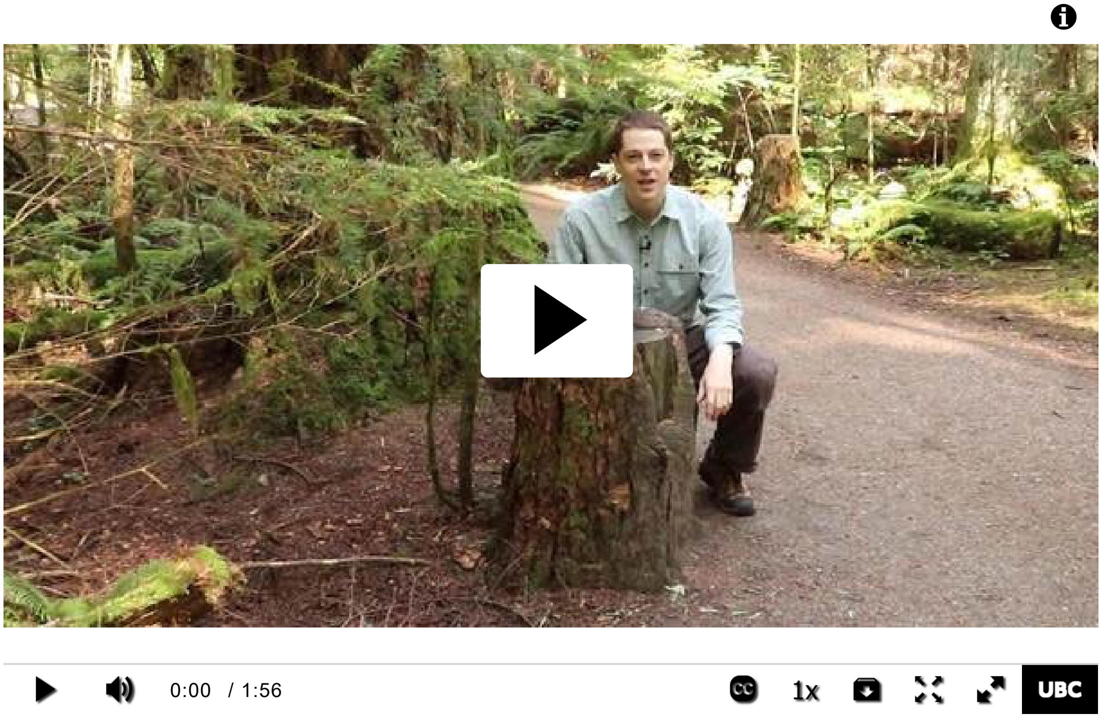

Springboard Notches

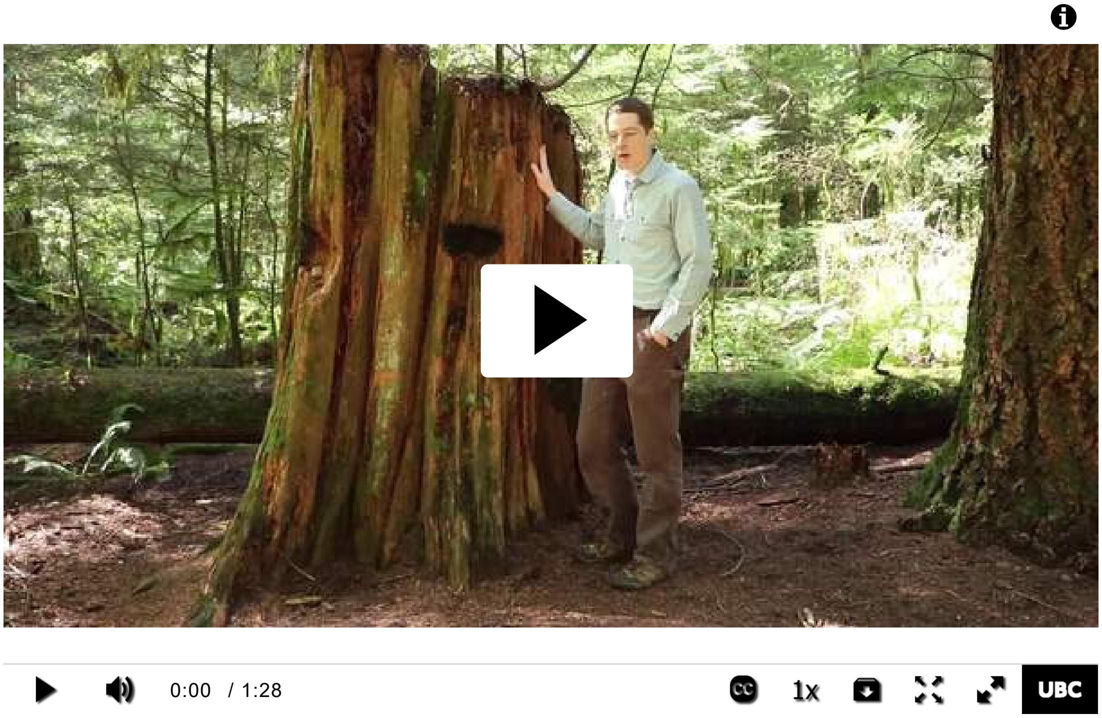

Nurse Log Regeneration (360 Video)

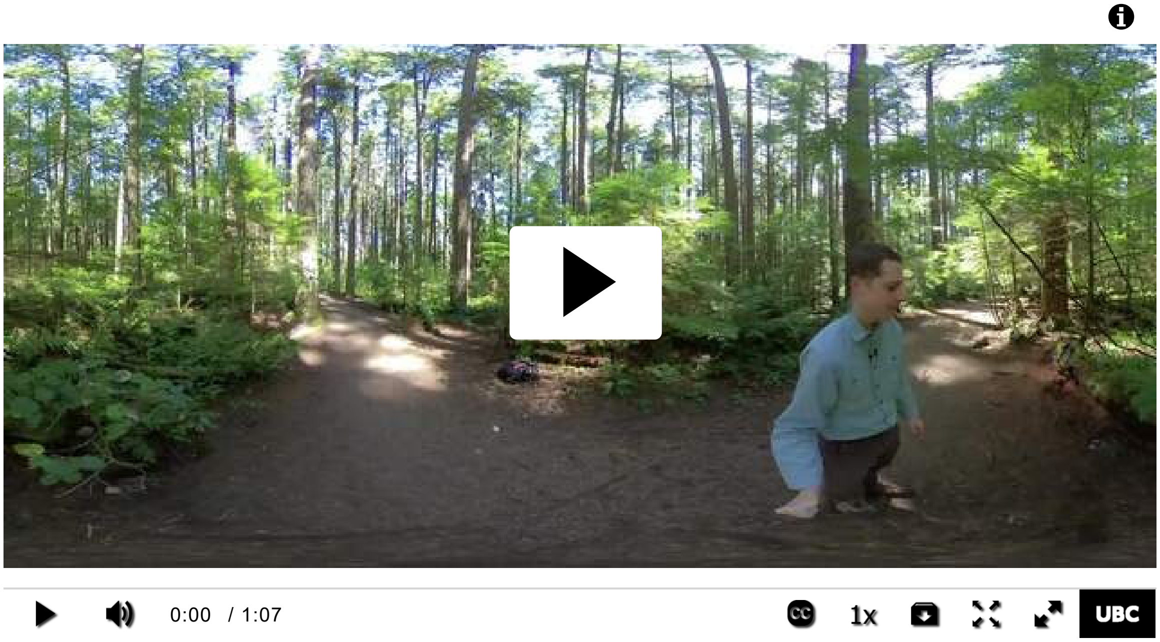

Nurse Log Regeneration - Advantages and Disadvantages

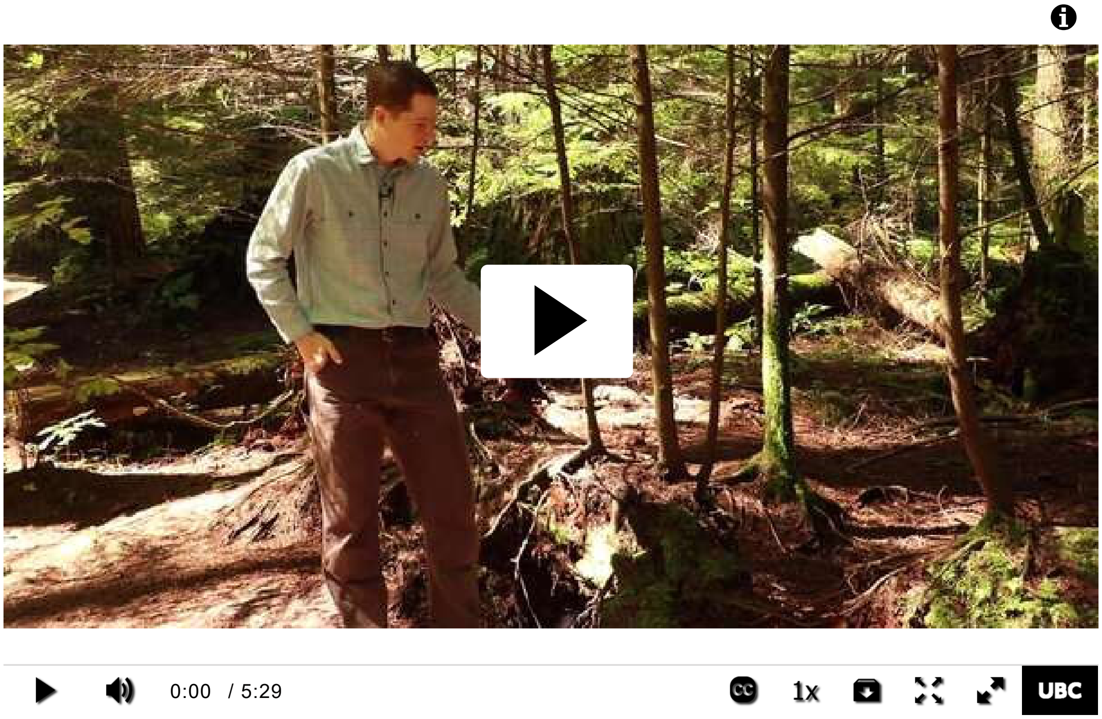

Gap Disturbance

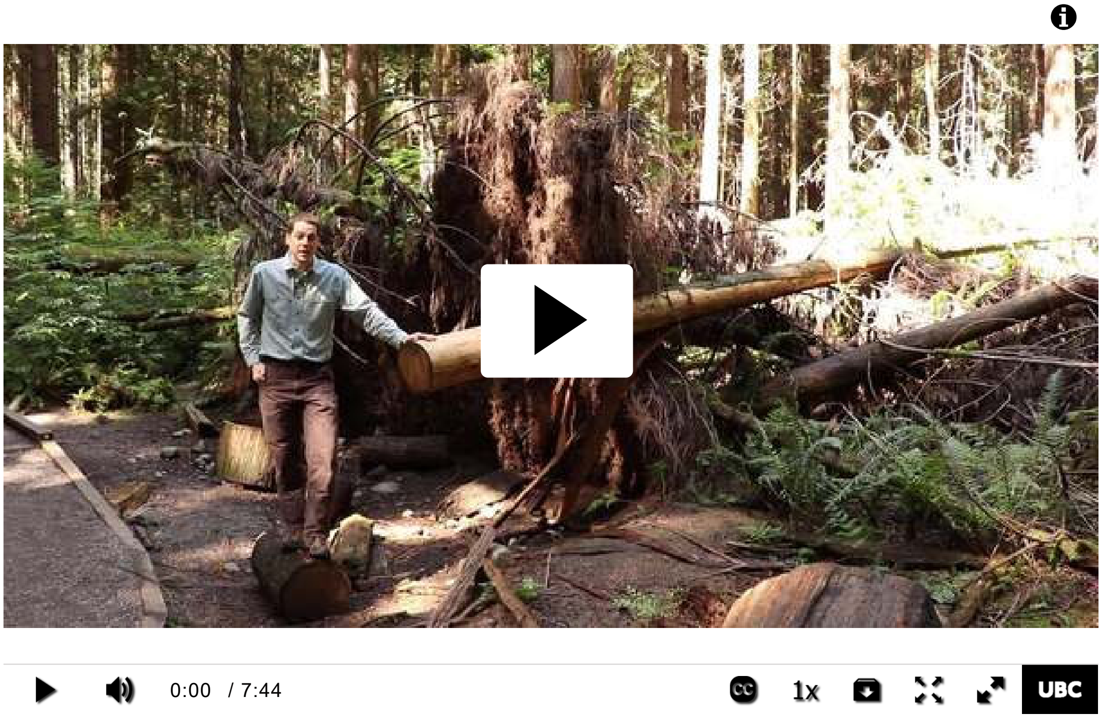

Gap Disturbance (360 Video)

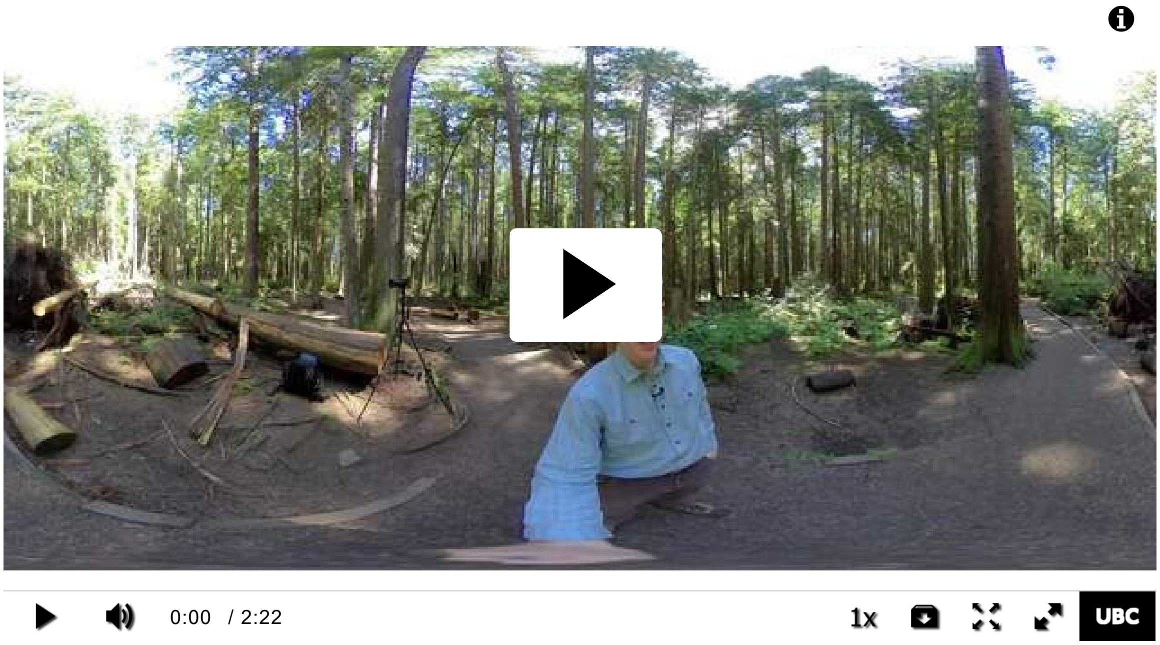

Wetter and Richer Sites

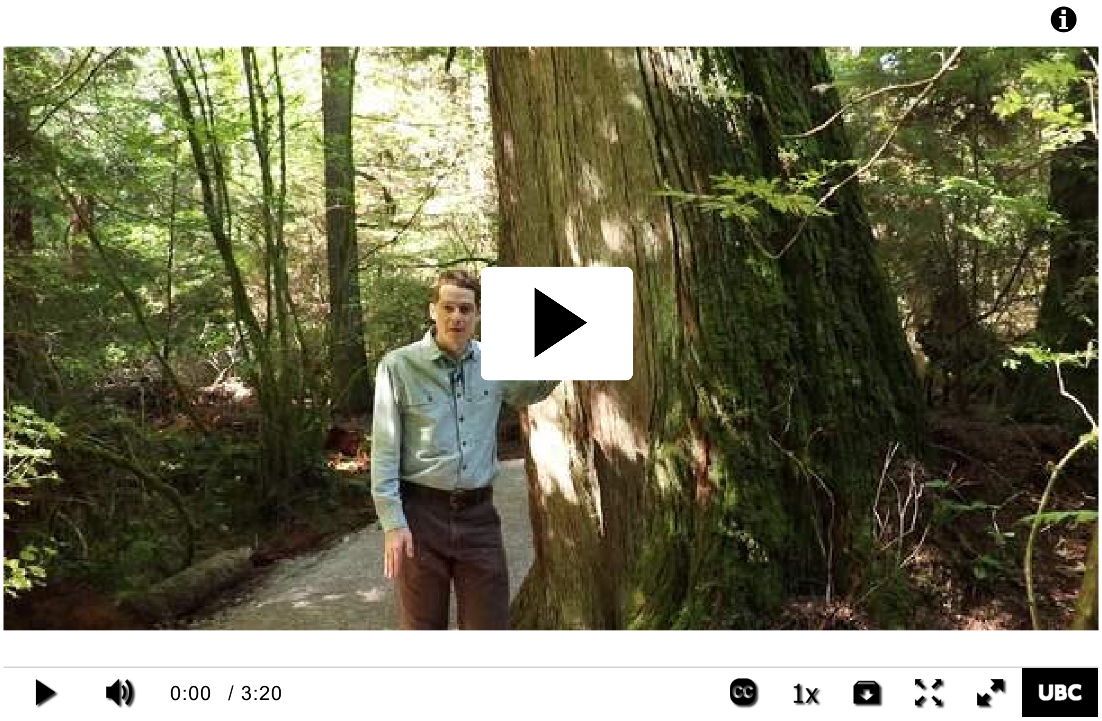

Pacific Spirit Park 360 Photo Tour

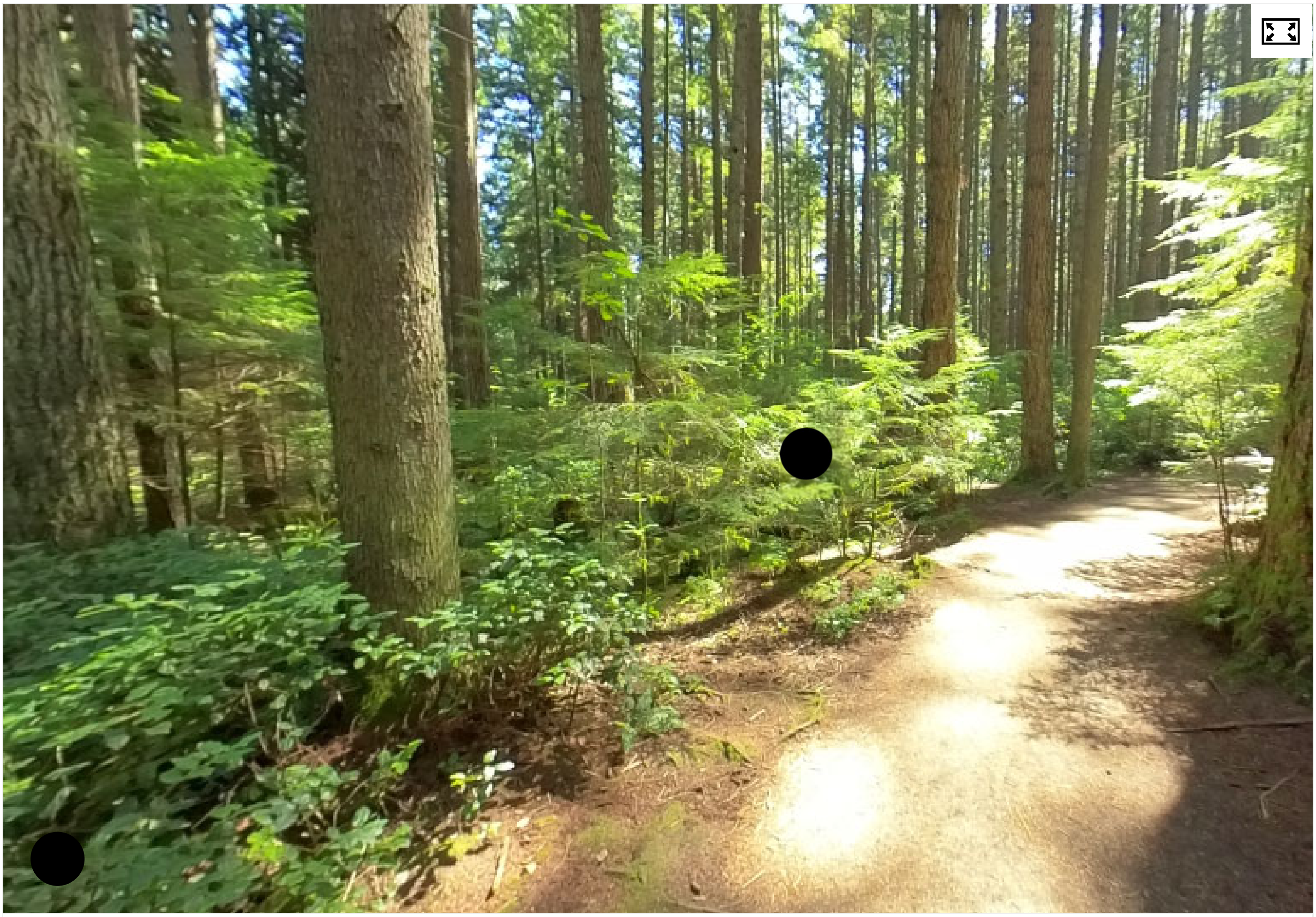

Reuse   <> Embed

Questions for Field Notebook:

1. Explain what a gap disturbance is?
2. How does a forest respond to a gap disturbance (what happens after the disturbance)?

Plant ID Walk

Part 1 (360 Video)

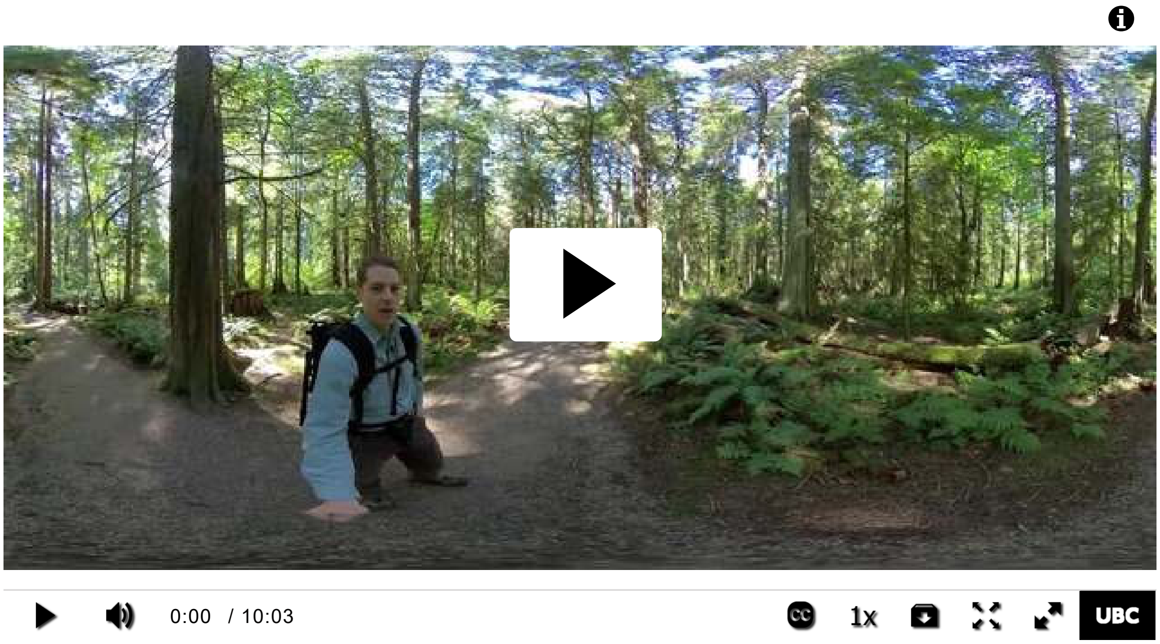

Part 2 (360 Video)

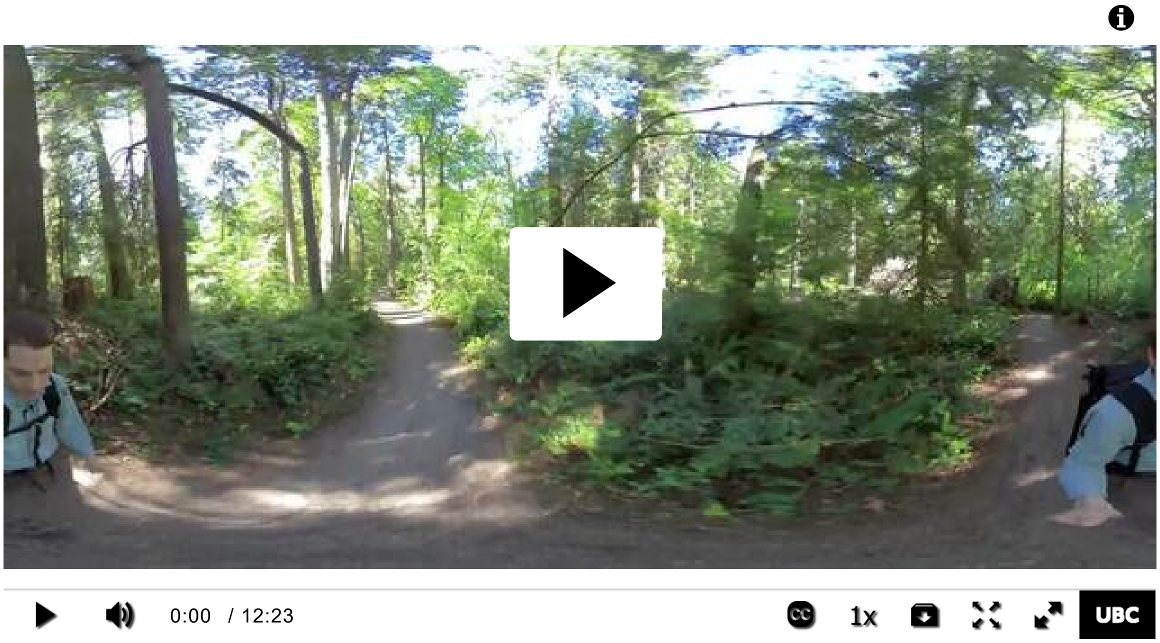

Part 3 (360 Video)

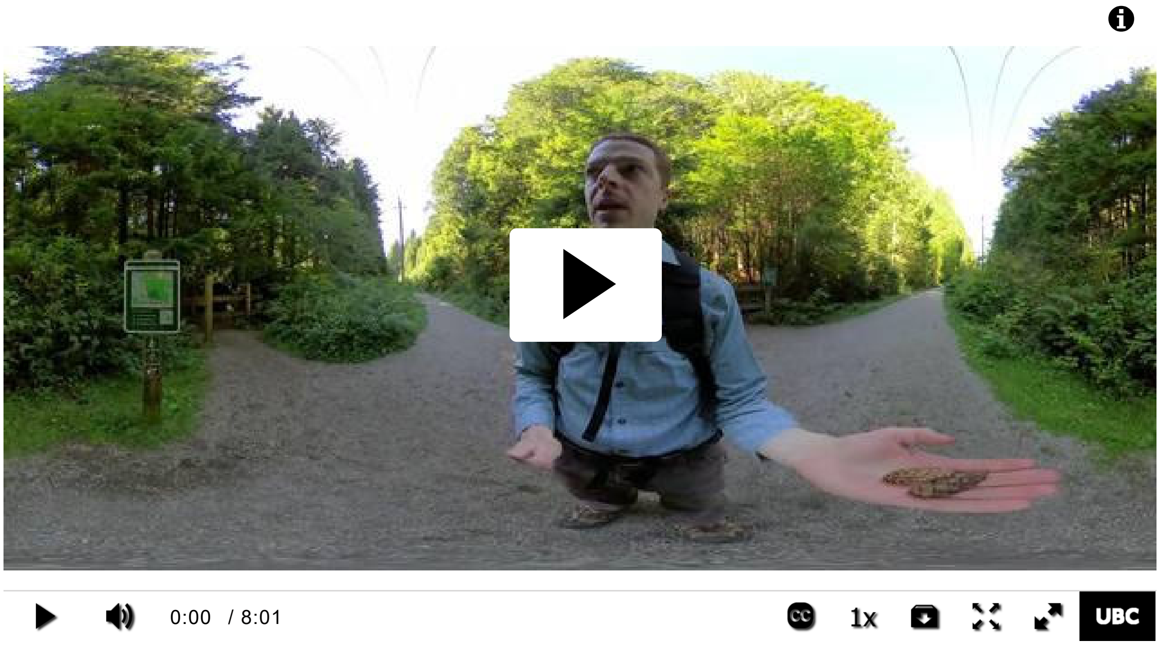
0:00 / 8:01
1x
UBC
