# Supplementary material for: COVID‐19 field instruction: Bringing the forests of British Columbia to students 8,000 km away
Source: Natural Sciences Education. 2021 Mar 10;50(1):e20040. doi: 10.1002/nse2.20040 (PMC7995167; doi:10.1002/nse2.20040)
Supplement: Supplementary file 2 — Supplementary material [file NSE2-50-e20040-s001.pdf]

Block 1

1. After reading the Survey consent form, do you consent to participating in this study? You have the right to refuse to participate in this study.

- Yes, I have read the survey consent form, and I consent to participating in this study.
- No. I DO NOT consent to participating in this study.

Default Question Block

2. My internet connection speed was adequate for viewing the **traditional (non-360°) videos.**

- Strongly agree
- Agree
- Somewhat agree
- Neither agree nor disagree
- Somewhat disagree
- Disagree
- Strongly disagree

3. My internet connection speed was adequate for viewing the **360° videos**.

- Strongly agree
- Agree
- Somewhat agree
- Neither agree nor disagree
- Somewhat disagree
- Disagree
- Strongly disagree

4. The **traditional (non-360°) videos** improved my understanding of the course material.

- Strongly agree
- Agree

- Somewhat agree
- Neither agree nor disagree
- Somewhat disagree
- Disagree
- Strongly disagree

5. The **360° still photographs** improved my understanding of the course material.

- Strongly agree
- Agree
- Somewhat agree
- Neither agree nor disagree
- Somewhat disagree
- Disagree
- Strongly disagree

6. The **360° videos** improved my understanding of the course material.

- Strongly agree
- Agree
- Somewhat agree
- Neither agree nor disagree
- Somewhat disagree
- Disagree
- Strongly disagree

7. The **traditional (non-360°) videos** and **360° still photographs** gave me the sensation of being in a forest.

- Strongly agree
- Agree
- Somewhat agree
- Neither agree nor disagree
- Somewhat disagree
- Disagree
- Strongly disagree

8. When viewing the **360° videos**, I was able to easily follow along and change my view to the object that Dr. Culbert was explaining.

- Strongly agree
- Agree
- Somewhat agree
- Neither agree nor disagree
- Somewhat disagree
- Disagree
- Strongly disagree

9. I frequently changed my view to look around while viewing **360° videos**.

- Strongly agree
- Agree
- Somewhat agree
- Neither agree nor disagree
- Somewhat disagree
- Disagree
- Strongly disagree

10. Did you use the subtitles (closed captions) while viewing the videos?

Yes	No
O	O

10. The video subtitles (closed captions) aided my understanding of the course material.

- Strongly agree
- Agree
- Somewhat agree
- Neither agree nor disagree
- Somewhat disagree
- Disagree
- Strongly disagree

11. How effective did you find each approach in improving your understanding of the course material? Please drag your mouse to sort the items below from most effective (1) to least effective (3).

Traditional videos

360° photographs

360° videos

12. Please explain why you ranked these approaches in this order.

13. How did you most often view the **traditional (non-360°) videos**?

- Streaming
- Downloading to computer first, then viewing local file.

14. How did you most often view the **360° videos**?

- Streaming
- Downloading to computer first, then viewing local file.

15. What type of device did you most often use to view course videos and 360° photographs?

- Phone
- Tablet
- Laptop computer
- Desktop computer

16. Did you have difficulty streaming, downloading, or viewing the videos or 360° photographs?

- Yes
- No

17. Please explain what happened.

18. From your perspective, what were the <u>advantages,</u> if any, of **traditional (non-360°) video**?

19. From your perspective, what were the <u>disadvantages,</u> if any, of **traditional (non-360°) video**?

20. From your perspective, what were the <u>advantages,</u> if any, of **360° photographs**?

21. From your perspective, what were the <u>disadvantages,</u> if any, of **360° photographs**?

22. From your perspective, what were the <u>advantages,</u> if any, of **360° video**?

23. From your perspective, what were the <u>disadvantages,</u> if any, of **360° video**?

24. What aspect(s) of this online field school did you like the <u>most?</u>

25. What aspect(s) of this online field school did you like the <u>least?</u>

26. How could this online field school be improved?

27. What advice would you give to instructors who will be teaching field courses in an online format?

Powered by Qualtrics
